# A global class reunion with multiple groups feasting on the declining insect smorgasbord

**DOI:** 10.1038/s41598-020-73609-9

**Published:** 2020-10-06

**Authors:** Eero J. Vesterinen, Kari M. Kaunisto, Thomas M. Lilley

**Affiliations:** 1 Department of Biology, University of Turku, Turku, Finland; 2 Department of Ecology, Swedish University of Agricultural Sciences, Uppsala, Sweden; 3 Biodiversity Unit, University of Turku, Turku, Finland; 4 Finnish Museum of Natural History, University of Helsinki, Helsinki, Finland

**Keywords:** Ecology, Evolution, Ecology, Environmental sciences

## Abstract

We report a detection of a surprising similarity in the diet of predators across distant phyla. Though just a first glimpse into the subject, our discovery contradicts traditional aspects of biology, as the earliest notions in ecology have linked the most severe competition of resources with evolutionary relatedness. We argue that our finding deserves more research, and propose a plan to reveal more information on the current biodiversity loss around the world. While doing so, we expand the recently proposed conservation roadmaps into a parallel study of global interaction networks.

## Introduction

Darwin suggested 150 years ago that competition is strongest among closely related species^1,2^. While this sentiment has endured time (but see^3,4^), it has been challenged by a call to increase attention to interactions between phylogenetically distant species^e.g.^^5^. Nowhere is this call more topical than in the context of interactions involving insect diet, together with the current worldwide decline in insect numbers and biomass. Where recent research has flagged impacts on mutualistic interactions (e.g. pollination^6^) as a particular concern, we know less about effects on antagonistic, trophic interactions. In this paper, we provide tentative evidence that predators separated by deep taxonomic divides (birds, bats, and dragonflies) tap into the very same resource setting of insect biomass, with widespread sharing of prey taxa. These findings suggest that the current insect decline may resonate widely across the tree of life and that a globally concerted effort to trace trophic links from insects across ecosystems is urgently needed.

Harvey et al*.*^7^ addressed a topical question regarding human-induced invertebrate loss^6, 8–11^. They formulated a global roadmap for insect conservation and hopeful recovery of endangered insect populations^7^. We wish to acknowledge the aforementioned prologue and to offer a complementary view to the biodiversity crisis around the planet. As the insects are declining, the insectivores are also in distress^12–16^. This leads to a notion that, instead of focusing solely on insects, we should simultaneously be studying interactions between insectivores and their prey^17^. Here we present the details of our discovery and propose a new, next-generation roadmap to concurrently disentangle the diversity of insect communities, their predators, and the resulting network.

Over the very last few decades, multiple studies have pointed to widespread decline among insects^7^. This pattern has been proposed to apply to both species-specific abundances and overall insect biomass and to extend across habitats with highly variable levels of human impact^6–11, 13,18^. This is alarming, given the dominance of insects in terms of diversity^19^ and biomass^20^, and the key role of insects in sustaining ecosystem processes and services^5,7^. The plight of pollinators has been of particular concern, considering its potential implications for plant pollination, crop production, and even vegetation composition^6^. But additionally, the overall insect decline will also affect the plethora of organisms utilizing insects as food. Recently, it has been suggested that many insectivores may be declining along with their diminishing prey^15^. A universal reduction of resources may both cause a decline in numbers of predators and change interaction dynamics between predators sharing their diet, commonly favoring generalists^6^.

To predict the consequences of the insect decline through trophic effects, we need to establish who eats whom and in what quantities. Very little is still known about the majority of interactions between the predators and their prey, in particular, we are lacking data on the diet of most insectivores, although they most likely form the largest guild of predators^21^. This is a glaring knowledge gap, seeing as both these prey and insectivore communities encompass the quantitatively dominant, most species-rich and economically crucial invertebrate organisms on Earth—such as Diptera, Lepidoptera, Coleoptera, and Hymenoptera—and vertebrate predators, bats and birds^20^. When combined, interactions between insect prey and insectivores may be dominant and at the same time most severely affected trophic interaction type of the Holocene^21^.

Where much work has been invested in retracing interactions between given taxa and particular insect prey, we argue that the real repercussions of insectivory can only be understood by a phylogenetically wide-spread examination of major insectivores—and for links among such taxa. Because multiple predators consume the same prey, quantifying the relative importance of each in each habitat is vital to understanding the net function of insectivory^17^. In this study, we offer the first preview into the combined food web of several distantly related insectivorous predators, discuss the importance of such synthesis, and offer a roadmap for future research.

## Material and methods

To disentangle the shared prey use by multiple predators in one region, we combined data from several studies focusing on insectivore-prey relationships as described below. We selected twelve species of insectivores from three guilds to assess the dietary similarity of these distantly related groups: European pied flycatcher *Ficedula hypoleuca* (Passeriformes, Sylvatidae) (Pallas, 1764) represents birds, Northern bat *Eptesicus nilssonii* (Keyserling & Blasius, 1839)*,* Brandt’s bat *M. brandtii* (Eversmann, 1845), whiskered bat *M. mystacinus* (Kuhl, 1817), Daubenton’s bat *Myotis daubentonii* (Kuhl, 1817)*,* and Brown long-eared bat *Plecotus auritus* (Linnaeus, 1758) (all species belong to the family Vespertilionidae) represent bats, and the northern bluet *Enallagma cyathigerum* (Charpentier, 1840), spearhead bluet *Coenagrion* hastulatum (Charpentier, 1825)*,* crescent bluet *C. lunulatum* (Charpentier, 1840), variable bluet *C. pulchellum* (Vander Linden, 1825) (Odonata, Coenagrionidae), common spreadwing *Lestes sponsa* (Hansemann, 1823) (Lestidae), and black darter *Sympetrum danae* (Sulzer, 1776) (Libellulidae) represent invertebrate aerial predators.

*Ficedula hypoleuca* chicks and adults were sampled during the summer 2014 in Southwestern Finland. The diet was analysed by molecular approach from faeces. Laboratory work closely followed^22^, with the details in the Supplemental information (Supplemental Text 1: Molecular analysis). The bat food web data in the current study was adopted from an earlier work, see Vesterinen et al.^23,24^ for details. Shortly, faecal DNA was extracted from bat droppings, and prey DNA was amplified using the same markers as *F. hypoleuca* samples above. Data for the damselflies and dragonflies in the current study was adopted from earlier works, see Kaunisto et al.^17,25,26^ and Vesterinen et al. ^27^ for details. For detailed bioinformatics, see Supplemental Text 2: Bioinformatics. All the dietary (bird, bat, and odonate) data were merged together in the subsequent analysis (for full prey species list, see Supplemental Text 3: Additional results, Table S1). We calculated the percent of occurrence (POO) by scaling the frequency of each prey item so that the sum across all food items was 100% following^28^. To visualize the trophic interactions, we used package *bipartite*^29^ implemented in program R^30^. We constructed an interaction web for three levels of prey, species, families, and orders, and highlighted the most common prey taxa (with at least 20% proportion of total frequencies). We analysed sampling adequacy and comparability of the datasets by calculating accumulation curves for each predator using the number of individuals and read counts using function *specaccum* in R package *vegan*^31^.

## Results

**Figure 1:**
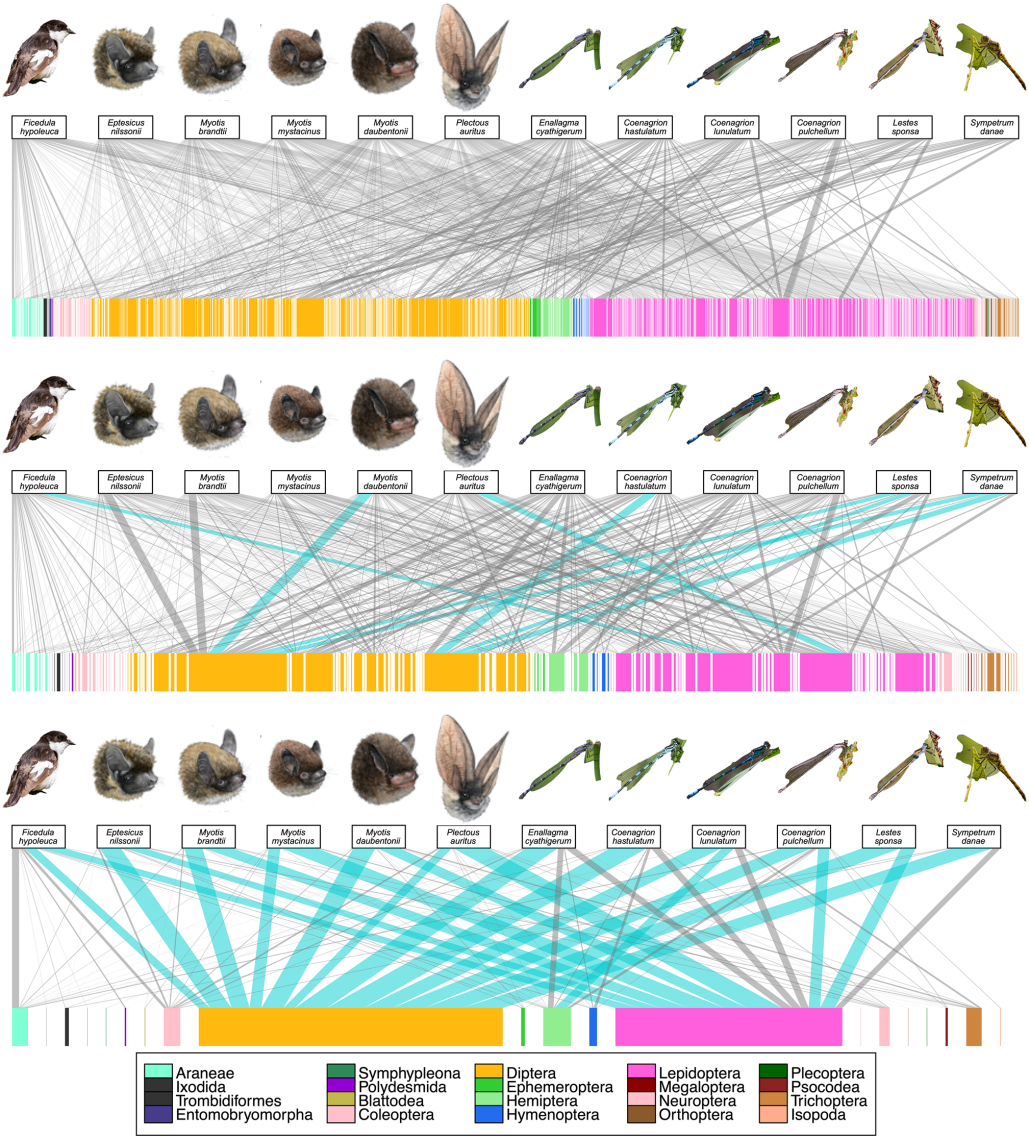
Food webs of twelve different insectivorous predator species (representing three different guilds) and their prey. The diets of three predator guilds: a bird, bats, and dragonflies sensu lato (including all odonates) are compared at three levels of taxonomic resolution: the prey species (top), the prey family (middle), and the prey order (bottom). The pictures in the upper row represent predators in each web and the blocks in the lower row the prey species. The prey is coloured by taxonomic order, as illustrated in the legend below the web. A line connecting a predator with a prey represents a predation record detected by molecular tools, and the thickness of the line represents the relative proportion of each predation record. For each predator, the prey interactions that correspond to at least 20% of the total diet are highlighted as turquoise. For details on methods and original data, including full prey species list, see Supplemental Text 3: Additional results. Photo credits: Maija Laaksonen (bat drawings), Kari Kaunisto (bird and dragonfly pictures).

**Figure 2:**
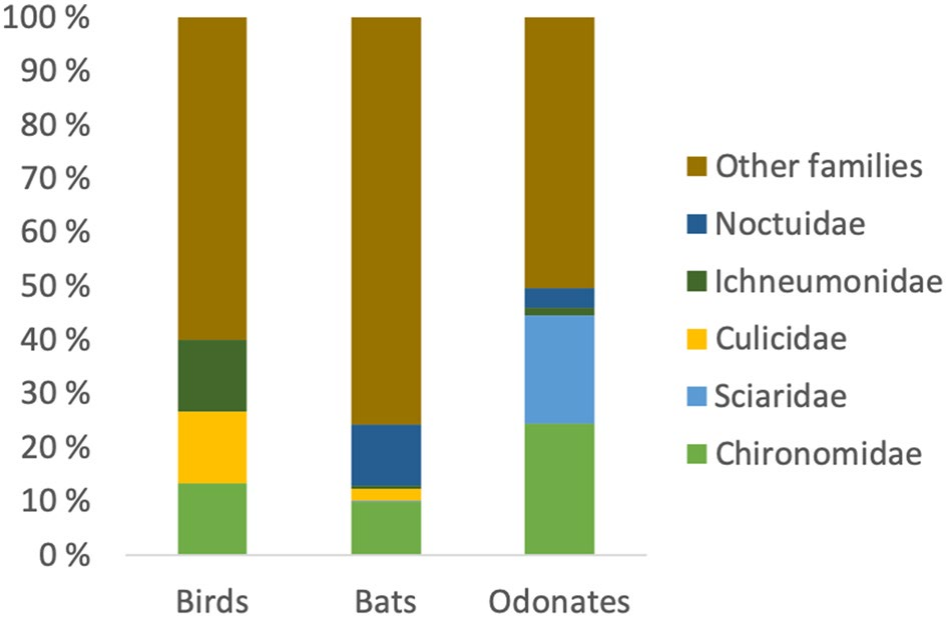
Prey use at the family level. Shown are the frequencies of the five most common families (DIPTERA: Chironomidae, Sciaridae, Culicidae; HYMENOPTERA: Ichneumonidae; and LEPIDOPTERA: Noctuidae) and of other families combined in the diet of each predator group. Chironomids are the biggest prey group for all of the insectivorous predators.

We identified altogether 924 prey taxa in 12 distinct predator species (see Supplemental Text 3: Additional results, Table S1, Figure S2, Figure S3, and Figure S4). Over 500 of these were shared by at least two predators and nearly two hundred by at least four predators. Two species (Lepidoptera, Gelechiidae, *Psoricoptera gibbosella* and Psocodea, Caeciliusidae, *Valenzuela flavidus*) were shared by all three guilds, and 64 more by at least two guilds. Insectivorous predators mainly used the arthropod orders Diptera and Lepidoptera in their diet, and the level of overlap increased towards the higher taxonomic level (from prey species to prey orders; Fig. 1). Chironomids (Diptera) were the most common prey group for all predators, and Sciaridae was another very important prey family (Fig. 2). At the prey order level, Diptera was found in all predator’s diet with at least 20% proportion of occurrences, and Lepidoptera was nearly as common (Fig. 1). The species accumulation curves based on read counts showed adequate sequencing depth (Figure S5), but the sampling seemed to have been somewhat limited (Figure S6).

**Figure 3:**
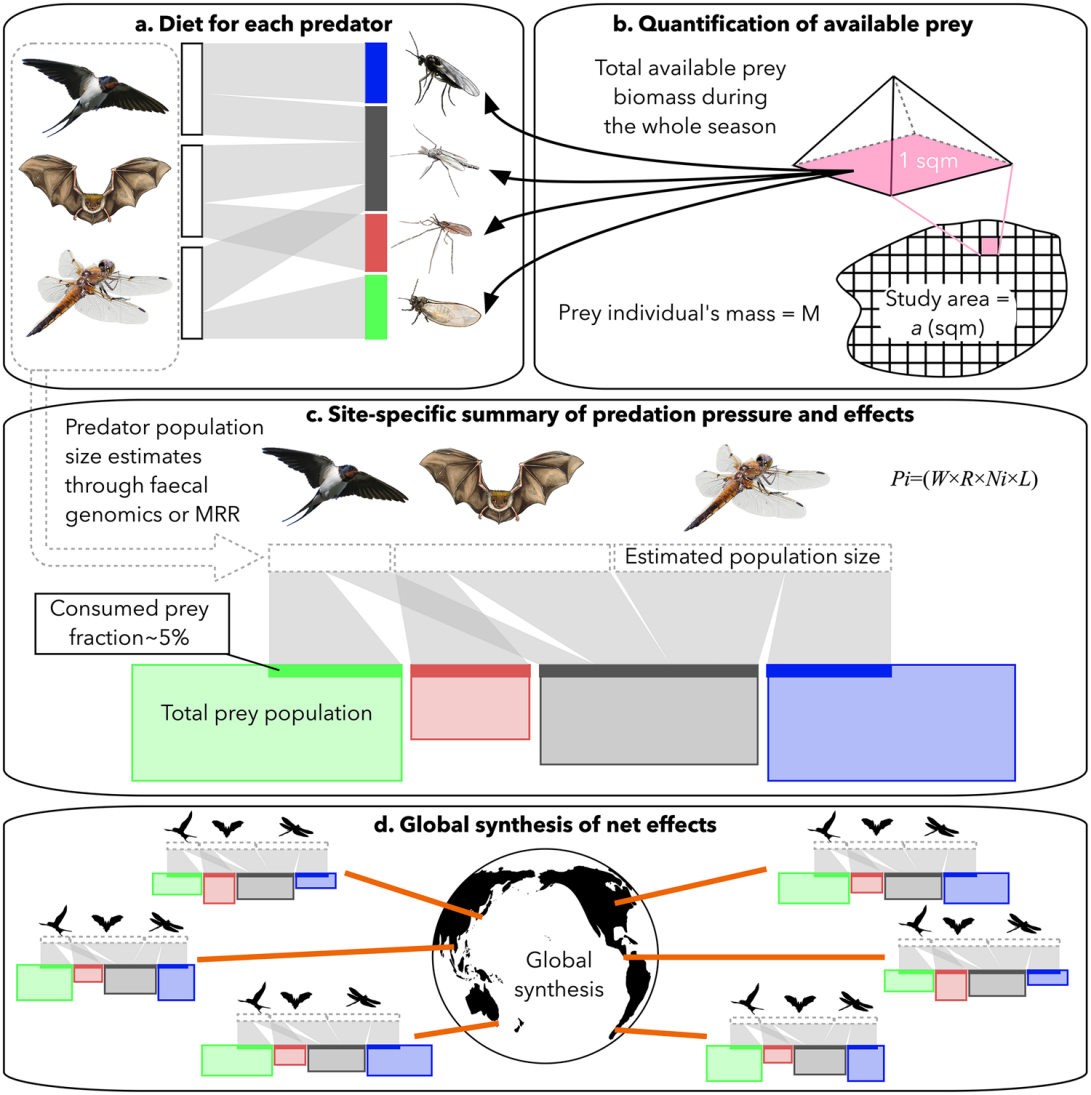
The roadmap to global insect-insectivore interactions and their potential effects on an ecosystem. To summarize the above-mentioned new focus, one needs to (**a**) describe the diet and measure population sizes for each predator. This can be achieved by applying calibrated molecular methods and relative read abundances (for RRA, see^28^). Predator population sizes may optimally be estimated directly from faecal DNA, using repeated sampling and population-genetic tools^56^. Secondly, one should (**b**) quantify the prey populations using area-standardized sampling methods, e.g. utilizing rearing traps and prey individual’s mass should be defined to estimate the total prey biomass. Thirdly, (**c**) these data should be combined with individual predator mass *W*, daily consumption rate *R*, predator population size *N*_*i*_, and the estimated longevity of each individual predator *L* in the following equation: *P*_*i*_ = (*W* × *R* × *N*_*i*_ × *L*) to calculate the species-specific consumption for each predator. For rationale and a detailed description of the equation, see^17^. Finally, (**d**) this pipeline should be replicated worldwide, to synthesize the global net effect for both predators and prey in the era of biodiversity crisis. Photo credits: Kari Kaunisto (bird picture in **a**,**c**), Anna Blomberg (bat drawing in **a**,**c**), Tuomas Kankaanpää (dragonfly picture in panels a and c prey insects in **a**,**c**), and Maija Laaksonen (bat silhouette in **d**). All other pictures by Eero Vesterinen.

As a roadmap for the assessment of insectivorous predators and their prey globally, we constructed a framework for future studies (Fig. 3). This includes the dissection of the diet for each predator (Fig. 3a), quantification of the available prey in each habitat (Fig. 3b), combining all the aforementioned data with predator population size estimates to end up with predation pressure summaries (Fig. 3c). Finally, we may construct a global synthesis of insectivore-prey interaction net effects (Fig. 3d).

## Discussion

In the current era of defaunation^32^, many organisms are declining rapidly^12,15,16,33–37^, and especially insects are suffering greatly^6,8–10,13,18,38,39^. This is alarming because we know very little of the fundamental interactions between the majority of species. With the current work, we offer the first evidence of a shared menu of distantly related predators, with the common denominator being their placement in the guild of insectivores (Figs. 1, 2). While reporting our findings, we also propose a roadmap for utilising the latest next-generation techniques to disentangle the global patterns of insectivory (Fig. 3). All the tools to accomplish this task are already available and in use, and can be combined with analytical protocols to build a novel synthesis, as outlined in Fig. 3.

Using state-of-the-art molecular tools for detecting trophic links, we report here a key finding: predator taxa, separated as far back as the phylum-level, share a major proportion of their prey taxa. This insight contradicts traditional wisdom^1^ because it implies that predators from different branches of the Tree of Life may interact with the same prey, and thus, either directly or indirectly with each other. This calls for a new approach to trophic biology: rather than applying focus solely on individual predators, predator orders, or even classes, we need to retrace interactions centered on similar prey throughout the ecosystem (Fig. 1). Only thereby can we understand the community-wide repercussions of a change in insect biomass and community composition.

As an example of potential outcomes of climate change, we pick a central prey group, non-biting midges (Fig. 2), which are widely used as bioindicators of water quality^40,41^. Aquatic chironomid larvae are characterised by synchronous mass-emergence, and their development is a function of temperature^42^. Thus, it is possible to envision a future scenario, where chironomids would either diminish (due to pollution of freshwater) or their mass-emergence phenology would shift (due to global warming). We should be asking; what kind of effects might arise in the aforementioned consequence? On one hand, the populations of insectivores depending on the lost resource would likely suffer and collapse^6^. This, on the other hand, could lead to a significant increase of harmful pest insects, profiting from a decrease in predation pressure^21^.

While our current results enable only a first glimpse of links between focal insectivores, we believe that they point to a general pattern—the details of which can only be filled in by a wider research programme targeting the global distribution of insectivory across taxa and regions (Fig. 3). Overall, we should simultaneously quantify the abundance of different predators, their diet, and the composition of available prey. While the proposed research scheme would have been considered as utopia just 10 years ago, the tools to accomplish this task are now readily available for all the focal groups^17, 22,25,43^. We can already accurately analyse the diet of various predators, including bats^22,23,44–46^, birds^43,47^, dragonflies and damselflies^17,25^, spiders^48,49^, and a variety of herbivores^50–52^. Also, surveying the available prey (i.e. any insects in the wild; Fig. 3b) is possible and sometimes more cost-efficient using molecular tools^22^. There are also many good suggestions for how to quantify insect biomasses (Fig. 3b), besides merely accounting for species richness^17, 53–55^. Predator identity can be retraced from faeces using DNA microsatellites^22^, and even the population size and density may be estimated directly from non-invasively collected samples^56^. In certain circumstances, however, it is more efficient to use mark-recapture methods to calculate effective predator population sizes^17^. When all of these variables are combined with predator-specific consumption, we may estimate the proportion of each prey population being consumed (Fig. 3c)^17^. Finally, such an approach could be repeated globally in various habitats, to reach a world-wide synthesis of the net effect of insectivory (Fig. 3d). To go deeper into ecological questions, it would be advisable to add analysis of functional diversity of prey, such as prey diversity indices, to receive a better understanding of how the ecosystem works^57–59^. By achieving this, we will reach a novel insight into the ecology of insectivores, how predators are linked through shared prey, and to which extent the declining populations of both might alter these interactions. Without high-quality quantitative information, it is difficult, maybe even impossible to estimate the effect of global changes on ecosystem-level dynamics.

## Conclusions

To conclude, we have proven that phylogenetically distinct insectivores may share a major proportion of their diet. Given the ecological and economical importance of insectivores globally, this topic deserves the attention of scientists throughout the world. To aid in this quest, we have planned a roadmap for researchers of various subjects. In our ideal scenario, the study organisms are not even captured or encountered, but instead, a set of traces (faeces, hair, etc.) are examined and used for detailed analysis. It is now time to put these modern methods to test through a simultaneous dissection of interactions among arthropod prey and multiple predator guilds across the world (Fig. 1). By following the roadmap outlined above (Fig. 3), we will reach a novel insight into the ecology of insectivores, how predators are intertwined through shared prey, and to which extent the declining populations of both the predators and the prey might alter these interactions. Without high-quality quantitative information, it is difficult, and maybe impossible, to estimate the consequences of the current insect decline on the ecosystem worldwide.

## Supplementary information

Supplementary Information.

## Data Availability

The data for this manuscript has already been published for bats^23, 24^ and dragonflies^25, 26^. The bird prey species list is provided in the online supplementary material and the sequence data will be published later in another article.

## References

[1] Darwin C (1859). On the Origin of Species.

[2] Gause GF (1934). The Struggle for Existence.

[3] Webb CO, Ackerly DD, McPeek MA, Donoghue MJ (2002). Phylogenies and community ecology. Annu. Rev. Ecol. Syst..

[4] Vamosi SM, Heard SB, Vamosi JC, Webb CO (2009). Emerging patterns in the comparative analysis of phylogenetic community structure. Mol. Ecol..

[5] Biere A, Bennett AE (2013). Three-way interactions between plants, microbes and insects. Funct. Ecol..

[6] Biesmeijer JC (2006). Parallel declines in pollinators and insect-pollinated plants in Britain and the Netherlands. Science.

[7] Harvey JA (2020). International scientists formulate a roadmap for insect conservation and recovery. Nat. Ecol. Evol..

[8] Hallmann CA (2017). More than 75 percent decline over 27 years in total flying insect biomass in protected areas. PLoS ONE.

[9] Leather SR (2018). “Ecological Armageddon”—more evidence for the drastic decline in insect numbers: Insect declines. Ann. Appl. Biol..

[10] Sánchez-Bayo F, Wyckhuys KAG (2019). Worldwide decline of the entomofauna: A review of its drivers. Biol. Conserv..

[11] Cardoso P (2020). Scientists’ warning to humanity on insect extinctions. Biol. Conserv..

[12] Ford HA, Barrett GW, Saunders DA, Recher HF (2001). Why have birds in the woodlands of Southern Australia declined?. Biol. Conserv..

[13] Córdoba-Aguilar A, Rocha-Ortega M (2019). Damselfly (Odonata: Calopterygidae) population decline in an urbanizing watershed. J. Insect Sci..

[14] Kalkman VJ (2018). Diversity and conservation of European dragonflies and damselflies (Odonata). Hydrobiologia.

[15] Rosenberg KV (2019). Decline of the North American avifauna. Science.

[16] Rodhouse TJ (2019). Evidence of region-wide bat population decline from long-term monitoring and Bayesian occupancy models with empirically informed priors. Ecol. Evol..

[17] Kaunisto KM (2020). Threats from the air: Damselfly predation on diverse prey taxa. J. Anim. Ecol..

[18] Simmons BI (2019). Worldwide insect declines: An important message, but interpret with caution. Ecol. Evol..

[19] Mora C, Tittensor DP, Adl S, Simpson AGB, Worm B (2011). How many species are there on earth and in the ocean?. PLoS Biol..

[20] Bar-On YM, Phillips R, Milo R (2018). The biomass distribution on earth. Proc. Natl. Acad. Sci..

[21] Nyffeler M, Şekercioğlu ÇH, Whelan CJ (2018). Insectivorous birds consume an estimated 400–500 million tons of prey annually. Sci. Nat..

[22] Vesterinen EJ (2016). What you need is what you eat? Prey selection by the bat *Myotis daubentonii*. Mol. Ecol..

[23] Vesterinen EJ, Puisto AIE, Blomberg A, Lilley TM (2018). Table for five, please: Dietary partitioning in boreal bats. Ecol. Evol..

[24] Vesterinen EJ, Puisto AIE, Blomberg AS, Lilley TM (2019). Data from: Table for five, please: Dietary partitioning in boreal bats. Dryad Dataset.

[25] Kaunisto KM, Roslin T, Sääksjärvi IE, Vesterinen EJ (2017). Pellets of proof: First glimpse of the dietary composition of adult odonates as revealed by metabarcoding of feces. Ecol. Evol..

[26] Kaunisto KM, Roslin TL, Sääksjärvi IE, Vesterinen EJ (2018). Data from: Pellets of proof: first glimpse of the dietary composition of adult odonates as revealed by metabarcoding of feces. Dryad Dataset.

[27] Vesterinen, E. J. *et al.*Threats from the air: damselfly predation on diverse prey taxa. 1438406240 bytes (2019) 10.5061/DRYAD.ZS7H44J4Z. 10.1111/1365-2656.13184 32124439

[28] Deagle BE (2019). Counting with DNA in metabarcoding studies: How should we convert sequence reads to dietary data?. Mol. Ecol..

[29] Dormann CF, Frund J, Bluthgen N, Gruber B (2009). Indices, graphs and null models: analyzing bipartite ecological networks. Open Ecol. J..

[30] R Core Team. *R: A Language and Environment for Statistical Computing*. (R Foundation for Statistical Computing, 2018).

[31] Oksanen, J. *et al. vegan: Community Ecology Package*. (2013).

[32] Dirzo R (2014). Defaunation in the anthropocene. Science.

[33] Butchart SHM (2010). Global biodiversity: Indicators of recent declines. Science.

[34] Fuszara E (2010). Population changes in Natterer’s bat (*Myotis nattereri*) and Daubenton’s bat (*M. daubentonii*) in winter roosts of central Poland. Pol. J. Ecol..

[35] Kim KC, Byrne LB (2006). Biodiversity loss and the taxonomic bottleneck: Emerging biodiversity science. Ecol. Res..

[36] Sekercioglu CH (2002). Disappearance of insectivorous birds from tropical forest fragments. Proc. Natl. Acad. Sci. USA..

[37] Spiller KJ, Dettmers R (2019). Evidence for multiple drivers of aerial insectivore declines in North America. Condor.

[38] Lister BC, Garcia A (2018). Climate-driven declines in arthropod abundance restructure a rainforest food web. Proc. Natl. Acad. Sci..

[39] Warren MS (2001). Rapid responses of British butterflies to opposing forces of climate and habitat change. Nature.

[40] Ochieng H, de Ruyter van Steveninck ED, Wanda FM (2008). Mouthpart deformities in Chironomidae (Diptera) as indicators of heavy metal pollution in northern Lake Victoria, Uganda. Afr. J. Aquat. Sci..

[41] Luoto TP (2010). Hydrological change in lakes inferred from midge assemblages through use of an intralake calibration set. Ecol. Monogr..

[42] *Aquatic insects of North Europe—A Taxonomic Handbook*. vol. 2 (Apollo Books, 1997).

[43] Wirta HK (2015). Exposing the structure of an Arctic food web. Ecol. Evol..

[44] Vesterinen EJ, Lilley T, Laine VN, Wahlberg N (2013). Next generation sequencing of fecal DNA reveals the dietary diversity of the widespread insectivorous predator Daubenton’s bat (*Myotis daubentonii*) in southwestern Finland. PLoS ONE.

[45] Clare EL, Fraser EE, Braid HE, Fenton MB, Hebert PDN (2009). Species on the menu of a generalist predator, the eastern red bat (*Lasiurus borealis*): Using a molecular approach to detect arthropod prey. Mol. Ecol..

[46] Clare EL (2014). The diet of *Myotis lucifugus* across Canada: Assessing foraging quality and diet variability. Mol. Ecol..

[47] Rytkönen S (2019). From feces to data: A metabarcoding method for analyzing consumed and available prey in a bird-insect food web. Ecol. Evol..

[48] Eitzinger B (2019). Assessing changes in arthropod predator–prey interactions through DNA-based gut content analysis—variable environment, stable diet. Mol. Ecol..

[49] Schmidt NM, Mosbacher JB, Eitzinger B, Vesterinen EJ, Roslin T (2018). High resistance towards herbivore-induced habitat change in a high Arctic arthropod community. Biol. Lett..

[50] Schmidt NM, Mosbacher JB, Vesterinen EJ, Roslin T, Michelsen A (2018). Limited dietary overlap amongst resident Arctic herbivores in winter: Complementary insights from complementary methods. Oecologia.

[51] Gripenberg S (2019). A highly resolved food web for insect seed predators in a species-rich tropical forest: Host use by insect seed predators. Ecol. Lett..

[52] Basset Y (2018). A cross-continental comparison of assemblages of seed- and fruit-feeding insects in tropical rain forests: Faunal composition and rates of attack. J. Biogeogr..

[53] Raitif J, Plantegenest M, Agator O, Piscart C, Roussel J-M (2018). Seasonal and spatial variations of stream insect emergence in an intensive agricultural landscape. Sci. Total Environ..

[54] Rogers LE, Buschbom RL, Watson CR (1977). Length-weight relationships of shrub-steppe invertebrates1. Ann. Entomol. Soc. Am..

[55] De Felici L, Piersma T, Howison RA (2019). Abundance of arthropods as food for meadow bird chicks in response to short- and long-term soil wetting in Dutch dairy grasslands. PeerJ.

[56] Aziz MA (2017). Using non-invasively collected genetic data to estimate density and population size of tigers in the Bangladesh Sundarbans. Glob. Ecol. Conserv..

[57] Greenop A, Woodcock BA, Wilby A, Cook SM, Pywell RF (2018). Functional diversity positively affects prey suppression by invertebrate predators: A meta-analysis. Ecology.

[58] Kissick AL, Dunning JB, Fernandez-Juricic E, Holland JD (2018). Different responses of predator and prey functional diversity to fragmentation. Ecol. Appl..

[59] Petchey OL, Gaston KJ (2006). Functional diversity: Back to basics and looking forward. Ecol. Lett..

