# Author Correction: A global class reunion with multiple groups feasting on the declining insect smorgasbord

**DOI:** 10.1038/s41598-021-87187-x

**Published:** 2021-04-01

**Authors:** Eero J. Vesterinen, Kari M. Kaunisto, Thomas M. Lilley

**Affiliations:** 1 Department of Biology, University of Turku, Turku, Finland; 2 Department of Ecology, Swedish University of Agricultural Sciences, Uppsala, Sweden; 3 Biodiversity Unit, University of Turku, Turku, Finland; 4 Finnish Museum of Natural History, University of Helsinki, Helsinki, Finland

Correction to: *Scientific Reports* 10.1038/s41598-020-73609-9, published online 06 October 2020

The Supplementary Information published with this Article contains errors.

In the section ‘Laboratory protocols for Odonata’, a reference and link are omitted.

In the section ‘Supplemental Text 2: Bioinformatics’, the raw sequence output for one of the datasets is missing, and the full script is missing.

Finally, the current version of the Supplementary Information mistakenly shows tracked changes.

The correct Supplementary Information file is provided below.

## Supplementary Information

Supplementary Information.

