# Supplementary material for: A global class reunion with multiple groups feasting on the declining insect smorgasbord
Source: Sci Rep. 2020 Oct 6;10:16595. doi: 10.1038/s41598-020-73609-9 (PMC7539006; doi:10.1038/s41598-020-73609-9)
Supplement: Supplementary file 1 — Supplementary Information. [file 41598_2020_73609_MOESM1_ESM.docx]

Supplemental information for

**A global class reunion with multiple groups feasting on the declining insect smorgasbord**

Eero J. Vesterinen, Kari M. Kaunisto & Thomas M. Lilley

Index

[Supplemental Text 1: Molecular analysis 1](#_Toc49290382)

[Laboratory protocols for Ficedula hypoleuca 1](#_Toc49290383)

[Laboratory protocols for bats 3](#_Toc49290384)

[Laboratory protocols for Odonata 3](#_Toc49290385)

[Supplemental Text 2: Bioinformatics 3](#_Toc49290386)

[Supplemental Text 3: Additional results 4](#_Toc49290387)

[Species accumulation curves 4](#_Toc49290388)

[Prey taxa lists 6](#_Toc49290389)

[Supplemental references 27](#_Toc49290390)

## Supplemental Text 1: Molecular analysis

### Laboratory protocols for *Ficedula hypoleuca*

*F. hypoleuca* (Passeriformes, Sylvatidae) chicks and adults were sampled during the summer 2014 in Southwestern Finland. Laboratory work closely followed ^1^, with the following details. Faecal DNA was extracted using QIAamp Fast DNA Stool Mini Kit (product nr 51604, Qiagen), according to the instructions for Pathogen detection (Quick Start Guide, version January 2014). To specifically amplify prey but not predator DNA, we chose the most common mitochondrial markers for molecular identification of animals: cytochrome oxidase subunit I (hereafter abbreviated as COI) and 16S ribosomal RNA (16S). To amplify suitable fragments of approximately same lengths, we applied two primer sets—COI: primers ZBJ-ArtF1c and ZBJ-ArtR2c after Zeale *et al.* ^2^ and 16S: primers Ins16S-1F and Ins16S-1Rshort after Clarke *et al.* ^3^. For each extract, we amplified the arthropod-specific locus with the following protocol: 2 µl of the template DNA was mixed with 0.375 µl of each tagged primer (final concentration 0.4 μM), 6.25 µl of 2x MyTaq RedMix (Bioline) and the reaction was filled up to 12.5 µl with double-distilled water. The PCR cycling conditions were as follows: 15 min in 95°C, then 35 cycles of 30 s in 95°C, 30 s in 50°C and 60 s in 72°C, ending with 10 min in 72°C. The primers were tagged by linker-tags to enable easy insertion of adapter in the subsequent PCR *(modified from Clarke et al.* ^4^). 2 µl of the PCR product was loaded to 2 % agarose gel and run at 95 V for 40 min. The successful samples were cleaned with 0.5 µl of Exonuclease I and 1.0 µl of FastAP Thermosensitive Alkaline Phosphatase (both from ThermoScientific), by heating to 37°C for 30 min and 85°C for 15 min.

5 µl of the cleaned PCR products were combined and used for a second PCR to attach IonTorrent-specific sequencing adapters and the sample-specific barcodes into the samples. Special, individually barcoded primers were used as follows: forward primers 5’-CCATCTCATCCCTGCGTGTCTCCGACTCAGxxxxxxxxxxGATacgacgttgtaaaa-3’ (x’s mark the place for sample-specific barcode sequence; linker-tag in small letters) and reverse primers 3’-linker-tagged P1-adapters 5’ CCTCTCTATGGGCAGTCGGTGATcattaagttcccatta-3’ (linker-tag in small letters). The protocol for adapter-PCR was as follows: for reaction volume of 12.5 µl, 7.625 µl distilled water, 0.25 µl KAPA HiFi DNA polymerase (1U/ µl, KAPA Biosystems, Wilmington, Massachusetts, USA), 2.5 µl 5X KAPA HiFi buffer and 0.375 µl 10mM KAPA dNTP Mix (both buffer and dNTP mix provided with the KAPA HiFi DNA polymerase), 0.3 µM forward primer, 0.3 µM reverse primer and 1 µl purified locus-specific PCR product. The PCR cycling conditions were 3 min in 95°C, then 35 cycles of 20 s in 98°C, 15 s in 60°C and 15 s in 72°C, ending with 1 min in 72°C.

2 µl of the adapter PCR product was loaded to a 2% agarose gel and run at 90 V for 45 min. 9 µl of the adapter PCR product was then cleaned with SPRI bead double purification (as described in Vesterinen *et al.* 2016), to discard un-specific PCR products longer than 400 bp and shorter than 200 bp, as follows: 6.75 µl SPRI bead solution was added to each sample, and mixed thoroughly by vortexing. The samples were incubated 5 min in room temperature to allow DNA to bind to the beads, after which the samples were placed on magnets. The supernatant (including only shorter than approx. 400 bp long DNA fragments) were transferred to a new plate, and 3.5 µl SPRI was added and the samples were vortexed. Again, the samples were incubated at RT for 5 min before placing them on magnet, after which the supernatant (including shorter than approx. 200 bp long DNA fragments) were discarded. The beads, to which the targeted length PCR-products were attached to, were then cleaned twice with 100 µl freshly made 80% ethanol and left to dry for approx. 20 min after the washes. 22 µl of purified and distilled water was then added to each sample, the samples were vortexed and placed on magnet, after which 20 µl of the supernatants with the cleaned PCR product were transferred to a new plate.

Purified adapter-PCR DNA concentrations were measured using Qubit Fluorometer, following the manufacturer’s instructions. Samples were then pooled in equimolar ratios. Pooled libraries were purified and concentrated using SPRI beads as above. The purified samples were loaded on a 1% Agarose gel, the 316 bp band was cut and purified using NucleoSpin Gel and PCR Clean-up kit according to manual (catalog number 740609, Macherey-Nagel, Düren, Germany). This combined DNA library was measured using BioAnalyzer 1600 (Agilent Technologies, Santa Clara, California, USA) and subsequently diluted into 26 pM. Sequencing on the Ion Torrent PGM platform (Life Technologies) was performed by Center of Evolutionary Applications, University of Turku, Finland. For clonal amplification of the DNA library we used the 200 bp template kit for Ion PGM One Touch 2, following the manufacturer’s instructions (Life Technologies, manual cat nr 4480974, Rev A.0). An extra wash step with OT2 wash solution was added at the end of the protocol. Template positive Ion Sphere Particles were enriched using the Ion One Touch ES. Sequencing was done on a 318 chip with Ion PGM Sequencing 200 Kit v2 according to standard protocol, using 500 flows (Life Technologies, manual cat nr 4482006, Rev 3.0). Torrent Suite 4.4.3 software was used for base calling and initial quality trimming. Sequencing was performed on the Ion Torrent PGM platform (Life Technologies) by the Center of Evolutionary Applications, University of Turku, Finland. The raw data will be made available by another publication.

### Laboratory protocols for bats

All the data in the current study was adopted from an earlier work, see Vesterinen *et al.* ^5,6^ for details. Shortly, the DNA was extracted from pooled dropping samples and amplified using primers ZBJ-ArtF1c and ZBJ-ArtR2c after Zeale *et al.* ^2^. The adapters and sample-specific indices were attached in the second PCR, after which all the samples were pooled, purified and subsequently sequenced at the Illumina MiSeq platform using v2 2x150bp sequencing kit. All the data is available at Dryad Digital Repository: <https://doi.org/10.5061/dryad.6880rf1>.

### Laboratory protocols for Odonata

All the data in the current study was adopted from an earlier works, see ^7–10^ for details. Shortly, the DNA was extracted from pooled dropping samples and primers ZBJ-ArtF1c and ZBJ-ArtR2c after Zeale *et al.* ^2^ and 16S: primers Ins16S-1F and Ins16S-1Rshort after Clarke *et al.* ^3^. The adapters and sample-specific indices were attached in the second PCR, after which all the samples were pooled, purified and subsequently sequenced at the Illumina MiSeq platform using v2 2x150bp sequencing kit. All the data is available at Dryad Digital Repository: <https://doi.org/10.5061/dryad.5n92p> and

## Supplemental Text 2: Bioinformatics

The bird data (2,142,827 raw reads) was uploaded to CSC servers ([www.csc.fi](http://www.csc.fi)) for processing. To retain comparability between different datasets in the current study (bats, bird, and dragonflies), only COI reads were used for this study, since the bat dietary data only had COI data. Raw sequences were splitted into individual samples using *fastq_strip_barcode_relabel2.py* python script (available from <https://drive5.com/python/summary.html>). The output was divided to forward and reverse reads, based on the 5’-end primer using custom *perl* script. Short reads (<100bp) were removed using FASTX Toolkit *fastx_clipper* command^11^. Primers and poor-quality regions were trimmed using *cutadapt* with 20% mismatch rate and 100bp minimum length^12^. Then, reverse reads were re-oriented using FASTX Toolkit *fastx_reverse_complement* command, and all reads were merged together. Reads were dereplicated with USEARCH *derep_fulllength* (minuniquesize 2), and finally clustered into OTUs at 97% threshold (= default) using full dynamic programming to find alignments with the maximum possible score using USEARCH^13^. The 1849 dereplicated reads were clustered into 210 OTUs (after 21 chimeras were removed). Readtable (a.k.a. otutable) was constructed with *uc2otutab.py* python script (available from <https://drive5.com/python/summary.html>). Most of the reads (98.8%; 21,969 reads) were mapped to the readtable. OTUs were assigned to species using custom-tailored bash script utilising BOLD APIs (on August 2020). Most of the OTUs matched reference database 100%, but all hits above 97% were accepted. A part of OTUs (37 OTUs with only 418 reads altogether) weren’t assigned, and were discarded. One assignation was corrected from *Ixodes cornuatus* to *I. ricinus* as it is the only *Ixodes* species living in the area. We calculated the frequency of each prey item across samples, and divided this with the sum of all occurrences to end up with per cent of occurrences (POO; ^14^). We merged Fichyp data with bat and dragonfly data, to end up with the final data table. One damselfly species, *Enallagma cyathigerum*, was present in both odonate datasets, and was merged into one for the plotweb, but analysed separately for the accumulation curves below. Altogether, we identified 924 prey taxa for the twelve predators.

## Supplemental Text 3: Additional results

### Prey taxa lists

The full list of prey species (Table S1, Figure S2), families (Figure S3), and orders (Figure S4) are given below.

Table S1. Full prey species taxa. The ID numbers correspond to prey species (lower boxes).

| ID | CLASS  Order | Family | Species |
| --- | --- | --- | --- |
|  | ARACHNIDA |  |  |
| 1 | Araneae | Anyphaenidae | *Anyphaena accentuata* |
| 2 | Araneae | Araneidae | *Araniella cucurbitina* |
| 3 | Araneae | Araneidae | *Larinioides patagiatus* |
| 4 | Araneae | Araneidae | *Nuctenea umbratica* |
| 5 | Araneae | Clubionidae | *Clubiona caerulescens* |
| 6 | Araneae | Clubionidae | *Clubiona comta* |
| 7 | Araneae | Clubionidae | *Clubiona lutescens* |
| 8 | Araneae | Clubionidae | *Clubiona pallidula* |
| 9 | Araneae | Gnaphosidae | *Gnaphosa bicolor* |
| 10 | Araneae | Gnaphosidae | *Gnaphosa muscorum* |
| 11 | Araneae | Gnaphosidae | *Haplodrassus soerenseni* |
| 12 | Araneae | Gnaphosidae | *Haplodrassus umbratilis* |
| 13 | Araneae | Linyphiidae | *Bolyphantes alticeps* |
| 14 | Araneae | Linyphiidae | *Diplostyla concolor* |
| 15 | Araneae | Linyphiidae | *Drapetisca socialis* |
| 16 | Araneae | Linyphiidae | *Neriene clathrata* |
| 17 | Araneae | Linyphiidae | *Neriene peltata* |
| 18 | Araneae | Linyphiidae | *Porrhomma convexum* |
| 19 | Araneae | Linyphiidae | *Tenuiphantes tenebricola* |
| 20 | Araneae | Linyphilidae | *Erigone psychrophila* |
| 21 | Araneae | Lycosidae | *Lycosidae sp.* |
| 22 | Araneae | Lycosidae | *Pardosa nigriceps* |
| 23 | Araneae | Lycosidae | *Xerolycosa nemoralis* |
| 24 | Araneae | Philodromidae | *Philodromus cespitum* |
| 25 | Araneae | Philodromidae | *Philodromus collinus* |
| 26 | Araneae | Philodromidae | *Philodromus fuscomarginatus* |
| 27 | Araneae | Philodromidae | *Philodromus margaritatus* |
| 28 | Araneae | Salticidae | *Heliophanus cupreus* |
| 29 | Araneae | Theridiidae | *Cryptachaea riparia* |
| 30 | Araneae | Theridiidae | *Dipoena braccata* |
| 31 | Araneae | Theridiidae | *Dipoena torva* |
| 32 | Araneae | Theridiidae | *Paidiscura pallens* |
| 33 | Araneae | Theridiidae | *Phylloneta sisyphia* |
| 34 | Araneae | Theridiidae | *Robertus lividus* |
| 35 | Araneae | Theridiidae | *Theridiidae sp.* |
| 36 | Araneae | Thomisidae | *Xysticus deichmanni* |
| 37 | Araneae | Thomisidae | *Xysticus lanio* |
| 38 | Araneae | Thomisidae | *Xysticus lanio* |
| 39 | Ixodida | Ixodidae | *Ixodes ricinus* |
| 40 | Trombidiformes | Eriophyidae | *Eriophyes sp. amu 499* |
| 41 | Trombidiformes | Eupoidae | *Eupoidae sp.* |
| 42 | Trombidiformes | Hygrobatidae | *Hygrobates longipalpis* |
|  | COLLEMBOLA |  |  |
| 43 | Entomobryomorpha | Entomobryidae | *Entomobrya marginata* |
| 44 | Entomobryomorpha | Isotomidae | *Anurophorus laricis* |
| 45 | Symphypleona | Bourletiellidae | *Deuterosminthurus sp* |
|  | DIPLOPODA |  |  |
| 46 | Polydesmida | Polydesmidae | *Polydesmus denticulatus* |
|  | INSECTA |  |  |
| 47 | Blattodea | Ectobiidae | *Ectobius sp.* |
| 48 | Coleoptera | Cantharidae | *Cantharidae sp.* |
| 49 | Coleoptera | Cantharidae | *Cantharis livida* |
| 50 | Coleoptera | Cantharidae | *Cantharis nigricans* |
| 51 | Coleoptera | Cantharidae | *Cantharis pellucida* |
| 52 | Coleoptera | Cantharidae | *Cantharis rufa* |
| 53 | Coleoptera | Cantharidae | *Podabrus alpinus* |
| 54 | Coleoptera | Cantharidae | *Rhagonycha lignosa* |
| 55 | Coleoptera | Carabidae | *Acupalpus parvulus* |
| 56 | Coleoptera | Carabidae | *Amara aenea* |
| 57 | Coleoptera | Carabidae | *Anchomenus dorsalis* |
| 58 | Coleoptera | Carabidae | *Badister bullatus* |
| 59 | Coleoptera | Carabidae | *Badister dilatatus* |
| 60 | Coleoptera | Carabidae | *Dromius fenestratus* |
| 61 | Coleoptera | Carabidae | *Pterostichus adstrictus* |
| 62 | Coleoptera | Carabidae | *Pterostichus melanarius* |
| 63 | Coleoptera | Carabidae | *Pterostichus nigrita* |
| 64 | Coleoptera | Carabidae | *Pterostichus sp.* |
| 65 | Coleoptera | Cerambycidae | *Acanthocinus aedilis* |
| 66 | Coleoptera | Cerambycidae | *Alosterna tabacicolor* |
| 67 | Coleoptera | Cerambycidae | *Monochamus urussovi* |
| 68 | Coleoptera | Chrysomelidae | *Psylliodes picinus* |
| 69 | Coleoptera | Coleoptera fam. | *Coleoptera sp.* |
| 70 | Coleoptera | Curculionidae | *Barypeithes pellucidus* |
| 71 | Coleoptera | Curculionidae | *Brachyderes incanus* |
| 72 | Coleoptera | Curculionidae | *Pissodes castaneus* |
| 73 | Coleoptera | Curculionidae | *Polydrusus pilosus* |
| 74 | Coleoptera | Curculionidae | *Strophosoma capitatum* |
| 75 | Coleoptera | Dytiscidae | *Colymbetes paykulli* |
| 76 | Coleoptera | Dytiscidae | *Hydaticus seminiger* |
| 77 | Coleoptera | Dytiscidae | *Hydroporus morio* |
| 78 | Coleoptera | Dytiscidae | *Ilybius ater* |
| 79 | Coleoptera | Dytiscidae | *Ilybius guttiger* |
| 80 | Coleoptera | Dytiscidae | *Laccophilus comes* |
| 81 | Coleoptera | Elateridae | *Ampedus balteatus* |
| 82 | Coleoptera | Elateridae | *Dalopius marginatus* |
| 83 | Coleoptera | Elateridae | *Denticollis linearis* |
| 84 | Coleoptera | Gyrinidae | *Gyrinus aeratus* |
| 85 | Coleoptera | Gyrinidae | *Orectochilus sp.* |
| 86 | Coleoptera | Gyrinidae | *Orectochilus villosus* |
| 87 | Coleoptera | Melyridae | *Dasytes plumbeus* |
| 88 | Coleoptera | Oedemeridae | *Calopus serraticornis* |
| 89 | Coleoptera | Oedemeridae | *Calopus sp.* |
| 90 | Coleoptera | Ptinidae | *Ptinus fur* |
| 91 | Coleoptera | Scirtidae | *Cyphon padi* |
| 92 | Coleoptera | Staphylinidae | *Dropephylla ioptera* |
| 93 | Coleoptera | Staphylinidae | *Nudobius lentus* |
| 94 | Coleoptera | Staphylinidae | *Quedius fuliginosus* |
| 95 | Coleoptera | Tenebrionidae | *Tenebrio molitor* |
| 96 | Diptera | Agromyzidae | *Phytomyza flavicornis* |
| 97 | Diptera | Anisopodidae | *Sylvicola cinctus* |
| 98 | Diptera | Anisopodidae | *Sylvicola fenestralis* |
| 99 | Diptera | Anthomyiidae | *Anthomyiidae* |
| 100 | Diptera | Anthomyiidae | *Anthomyiidae sp.* |
| 101 | Diptera | Anthomyiidae | *Delia florilega* |
| 102 | Diptera | Anthomyiidae | *Delia platura* |
| 103 | Diptera | Anthomyiidae | *Delia sp.* |
| 104 | Diptera | Anthomyiidae | *Hydrophoria lancifer* |
| 105 | Diptera | Anthomyiidae | *Pegomya* |
| 106 | Diptera | Anthomyiidae | *Pegomya rubivora* |
| 107 | Diptera | Anthomyiidae | *Pegomya sp. BOLD:ACR0704* |
| 108 | Diptera | Anthomyiidae | *Pegoplata annulata* |
| 109 | Diptera | Anthomyiidae | *Zaphne ambigua* |
| 110 | Diptera | Anthomyzidae | *Anthomyza* |
| 111 | Diptera | Asilidae | *Dioctria hyalipennis* |
| 112 | Diptera | Bibionidae | *Bibio nigriventris* |
| 113 | Diptera | Bibionidae | *Bibio varipes* |
| 114 | Diptera | Bolitophilidae | *Bolitophila cinerea* |
| 115 | Diptera | Calliphoridae | *Calliphora vicina* |
| 116 | Diptera | Calliphoridae | *Melinda viridicyanea* |
| 117 | Diptera | Calliphoridae | *Pollenia griseotomentosa* |
| 118 | Diptera | Cecidomyiidae | *CecidInt12 sp* |
| 119 | Diptera | Cecidomyiidae | *CecidInt26 sp* |
| 120 | Diptera | Cecidomyiidae | *CecidInt35 sp. BOLD:ACB9926* |
| 121 | Diptera | Cecidomyiidae | *CecidInt35 sp. BOLD:ACP6401* |
| 122 | Diptera | Cecidomyiidae | *Cecidomyiidae sp.* |
| 123 | Diptera | Cecidomyiidae | *Feltiella acarisuga* |
| 124 | Diptera | Cecidomyiidae | *Ozirhincus sp* |
| 125 | Diptera | Ceratopogonidae | *Forcipomyia nigrans* |
| 126 | Diptera | Ceratopogonidae | *Forcipomyia sp* |
| 127 | Diptera | Ceratopogonidae | *Palpomyia lineata* |
| 128 | Diptera | Ceratopogonidae | *Serromyia femorata* |
| 129 | Diptera | Chaoboridae | *Chaoborus flavicans* |
| 130 | Diptera | Chaoboridae | *Chaoborus sp. BOLD:AAG5462* |
| 131 | Diptera | Chaoboridae | *Mochlonyx velutinus* |
| 132 | Diptera | Chironomidae | *Ablabesmyia aspera* |
| 133 | Diptera | Chironomidae | *Ablabesmyia longistyla* |
| 134 | Diptera | Chironomidae | *Ablabesmyia monilis* |
| 135 | Diptera | Chironomidae | *Ablabesmyia sp.* |
| 136 | Diptera | Chironomidae | *Arctopelopia barbitarsis* |
| 137 | Diptera | Chironomidae | *Brillia longifurca* |
| 138 | Diptera | Chironomidae | *Chaetocladius perennis* |
| 139 | Diptera | Chironomidae | *Chironomidae sp.* |
| 140 | Diptera | Chironomidae | *Chironomidae sp. BOLD:ACP1316* |
| 141 | Diptera | Chironomidae | *Chironomidae sp. BOLD:ACP6873* |
| 142 | Diptera | Chironomidae | *Chironomidae sp. BOLD:ACQ8800* |
| 143 | Diptera | Chironomidae | *Chironomidae sp. BOLD:ACU9532* |
| 144 | Diptera | Chironomidae | *Chironominae sp.* |
| 145 | Diptera | Chironomidae | *Chironomus acidophilus* |
| 146 | Diptera | Chironomidae | *Chironomus pseudomendax* |
| 147 | Diptera | Chironomidae | *Chironomus sp. BOLD:AAI4299* |
| 148 | Diptera | Chironomidae | *Chironomus sp. BOLD:AAI4301* |
| 149 | Diptera | Chironomidae | *Chironomus sp.1* |
| 150 | Diptera | Chironomidae | *Chironomus sp.2* |
| 151 | Diptera | Chironomidae | *Cladopelma sp.* |
| 152 | Diptera | Chironomidae | *Cladopelma sp. 1TE* |
| 153 | Diptera | Chironomidae | *Conchapelopia melanops* |
| 154 | Diptera | Chironomidae | *Conchapelopia sp. AJ-2011* |
| 155 | Diptera | Chironomidae | *Conchapelopia sp. BOLD:ACQ3496* |
| 156 | Diptera | Chironomidae | *Corynoneura scutellata* |
| 157 | Diptera | Chironomidae | *Cricotopus bicinctus* |
| 158 | Diptera | Chironomidae | *Cricotopus sp.* |
| 159 | Diptera | Chironomidae | *Cricotopus sylvestris* |
| 160 | Diptera | Chironomidae | *Cricotopus triannulatus* |
| 161 | Diptera | Chironomidae | *Cryptochironomus supplicans* |
| 162 | Diptera | Chironomidae | *Cryptochironomus supplicans* |
| 163 | Diptera | Chironomidae | *Demicryptochironomus sp.* |
| 164 | Diptera | Chironomidae | *Dicrotendipes lobiger* |
| 165 | Diptera | Chironomidae | *Dicrotendipes modestus* |
| 166 | Diptera | Chironomidae | *Dicrotendipes nervosus* |
| 167 | Diptera | Chironomidae | *Dicrotendipes tritomus* |
| 168 | Diptera | Chironomidae | *Einfeldia synchrona* |
| 169 | Diptera | Chironomidae | *Endochironomus tendens* |
| 170 | Diptera | Chironomidae | *Eukiefferiella dittmari* |
| 171 | Diptera | Chironomidae | *Glyptotendipes barbipes* |
| 172 | Diptera | Chironomidae | *Glyptotendipes cauliginellus* |
| 173 | Diptera | Chironomidae | *Glyptotendipes glacus* |
| 174 | Diptera | Chironomidae | *Glyptotendipes lobiferus* |
| 175 | Diptera | Chironomidae | *Glyptotendipes mancunianus* |
| 176 | Diptera | Chironomidae | *Glyptotendipes pallens* |
| 177 | Diptera | Chironomidae | *Glyptotendipes sp.* |
| 178 | Diptera | Chironomidae | *Heterotrissocladius marcidus* |
| 179 | Diptera | Chironomidae | *Kiefferulus sp.* |
| 180 | Diptera | Chironomidae | *Limnophyes sp* |
| 181 | Diptera | Chironomidae | *Metriocnemus fuscipes* |
| 182 | Diptera | Chironomidae | *Metriocnemus sp. 3ES* |
| 183 | Diptera | Chironomidae | *Microchironomus tener* |
| 184 | Diptera | Chironomidae | *Microtendipes chloris* |
| 185 | Diptera | Chironomidae | *Microtendipes pedellus* |
| 186 | Diptera | Chironomidae | *Microtendipes sp.* |
| 187 | Diptera | Chironomidae | *Orthocladiinae sp.* |
| 188 | Diptera | Chironomidae | *Parachironomus digitalis* |
| 189 | Diptera | Chironomidae | *Parachironomus monochromus* |
| 190 | Diptera | Chironomidae | *Paracladopelma* |
| 191 | Diptera | Chironomidae | *Paracladopelma sp.* |
| 192 | Diptera | Chironomidae | *Parakiefferiella sp.* |
| 193 | Diptera | Chironomidae | *Paratanytarsus dissimilis* |
| 194 | Diptera | Chironomidae | *Paratanytarsus laccophilus* |
| 195 | Diptera | Chironomidae | *Phaenopsectra punctipes* |
| 196 | Diptera | Chironomidae | *Polypedilum* |
| 197 | Diptera | Chironomidae | *Polypedilum convictum* |
| 198 | Diptera | Chironomidae | *Polypedilum nubeculosum* |
| 199 | Diptera | Chironomidae | *Polypedilum pedestre* |
| 200 | Diptera | Chironomidae | *Polypedilum sordens* |
| 201 | Diptera | Chironomidae | *Polypedilum sp. 1* |
| 202 | Diptera | Chironomidae | *Polypedilum sp. 2* |
| 203 | Diptera | Chironomidae | *Polypedilum sp. BOLD:ACR0701* |
| 204 | Diptera | Chironomidae | *Procladius cf. cinereus* |
| 205 | Diptera | Chironomidae | *Procladius cf. flavifrons* |
| 206 | Diptera | Chironomidae | *Procladius crassinervis* |
| 207 | Diptera | Chironomidae | *Procladius culiciformis* |
| 208 | Diptera | Chironomidae | *Procladius ferrugineus* |
| 209 | Diptera | Chironomidae | *Procladius nigriventris* |
| 210 | Diptera | Chironomidae | *Procladius sp. 1* |
| 211 | Diptera | Chironomidae | *Procladius sp. 1ES* |
| 212 | Diptera | Chironomidae | *Procladius sp. 2* |
| 213 | Diptera | Chironomidae | *Psectrocladius limbatellus* |
| 214 | Diptera | Chironomidae | *Psectrocladius octomaculatus* |
| 215 | Diptera | Chironomidae | *Psectrocladius schlienzi* |
| 216 | Diptera | Chironomidae | *Psectrocladius sp.* |
| 217 | Diptera | Chironomidae | *Psectrotanypus varius* |
| 218 | Diptera | Chironomidae | *Pseudosmittia trilobata* |
| 219 | Diptera | Chironomidae | *Rheotanytarsus pentapoda* |
| 220 | Diptera | Chironomidae | *Rheotanytarsus sp.* |
| 221 | Diptera | Chironomidae | *Smittia edwardsi* |
| 222 | Diptera | Chironomidae | *Stictochironomus sp. 3TE* |
| 223 | Diptera | Chironomidae | *Synendotendipes impar* |
| 224 | Diptera | Chironomidae | *Tanytarsus eminulus* |
| 225 | Diptera | Chironomidae | *Tanytarsus mendax* |
| 226 | Diptera | Chironomidae | *Thienemannimyia carnea* |
| 227 | Diptera | Chironomidae | *Xenochironomus xenolabis* |
| 228 | Diptera | Chironomidae | *Zavrelimyia melanura* |
| 229 | Diptera | Chironomidae | *Zavrelimyia sp.* |
| 230 | Diptera | Chloropidae | *Thaumatomyia* |
| 231 | Diptera | Chloropidae | *Thaumatomyia notata* |
| 232 | Diptera | Chloropidae | *Thaumatomyia sp. BOLD:ACX2752* |
| 233 | Diptera | Culicidae | *Aedes cataphylla* |
| 234 | Diptera | Culicidae | *Aedes cinereus* |
| 235 | Diptera | Culicidae | *Aedes communis* |
| 236 | Diptera | Culicidae | *Aedes hexodontus* |
| 237 | Diptera | Culicidae | *Aedes vexans* |
| 238 | Diptera | Culicidae | *Anopheles claviger* |
| 239 | Diptera | Culicidae | *Anopheles messeae* |
| 240 | Diptera | Culicidae | *Coquillettidia nigricans* |
| 241 | Diptera | Culicidae | *Culex orientalis* |
| 242 | Diptera | Culicidae | *Culex pipiens* |
| 243 | Diptera | Culicidae | *Culicidae sp.* |
| 244 | Diptera | Culicidae | *Culicidae sp. LAMV_M07_contig4* |
| 245 | Diptera | Culicidae | *Culiseta annulata* |
| 246 | Diptera | Culicidae | *Culiseta morsitans* |
| 247 | Diptera | Culicidae | *Culiseta nipponica* |
| 248 | Diptera | Culicidae | *Ochlerotatus cantans* |
| 249 | Diptera | Culicidae | *Ochlerotatus cataphylla* |
| 250 | Diptera | Culicidae | *Ochlerotatus excrucians* |
| 251 | Diptera | Culicidae | *Ochlerotatus intrudens* |
| 252 | Diptera | Culicidae | *Ochlerotatus punctor* |
| 253 | Diptera | Diptera fam. | *Diptera sp.* |
| 254 | Diptera | Dolichopodidae | *Dolichopus trivialis* |
| 255 | Diptera | Dolichopodidae | *Gymnopternus sp.* |
| 256 | Diptera | Dolichopodidae | *Hydrophorus praecox* |
| 257 | Diptera | Dolichopodidae | *Neurigona quadrifasciata* |
| 258 | Diptera | Drosophilidae | *Drosophilidae* |
| 259 | Diptera | Drosophilidae | *Scaptomyza pallida* |
| 260 | Diptera | Empididae | *Empis sp.* |
| 261 | Diptera | Empididae | *Empis tessellata* |
| 262 | Diptera | Empididae | *Hilara quadrifasciata* |
| 263 | Diptera | Empididae | *Rhamphomyia anaxo* |
| 264 | Diptera | Empididae | *Rhamphomyia caesia* |
| 265 | Diptera | Empididae | *Rhamphomyia nigripennis* |
| 266 | Diptera | Empididae | *Rhamphomyia nigrita* |
| 267 | Diptera | Empididae | *Rhamphomyia nr. anaxo* |
| 268 | Diptera | Empididae | *Rhamphomyia poplitea* |
| 269 | Diptera | Empididae | *Rhamphomyia sp.* |
| 270 | Diptera | Empididae | *Rhamphomyia umbripennis* |
| 271 | Diptera | Empididae | *Rhamphomyia valga* |
| 272 | Diptera | Fanniidae | *Fannia corvina* |
| 273 | Diptera | Fanniidae | *Fannia minutipalpis* |
| 274 | Diptera | Fanniidae | *Fannia sociella* |
| 275 | Diptera | Fanniidae | *Fannia sp.* |
| 276 | Diptera | Heleomyzidae | *Neoleria ruficeps* |
| 277 | Diptera | Heleomyzidae | *Suillia bicolor* |
| 278 | Diptera | Hippoboscidae | *Nycteribia kolenati* |
| 279 | Diptera | Hybotidae | *Bicellaria simplicipes* |
| 280 | Diptera | Hybotidae | *Euthyneura myrtilli* |
| 281 | Diptera | Hybotidae | *Leptopeza nr. borealis* |
| 282 | Diptera | Hybotidae | *Platypalpus harpiger* |
| 283 | Diptera | Hybotidae | *Platypalpus tuomikoskii* |
| 284 | Diptera | Hybotidae | *Trichina elongata* |
| 285 | Diptera | Keroplatidae | *Macrocera parva* |
| 286 | Diptera | Keroplatidae | *Macrocera stigma* |
| 287 | Diptera | Limoniidae | *Austrolimnophila unica* |
| 288 | Diptera | Limoniidae | *Dicranomyia cf. mitis* |
| 289 | Diptera | Limoniidae | *Dicranomyia didyma* |
| 290 | Diptera | Limoniidae | *Dicranomyia frontalis* |
| 291 | Diptera | Limoniidae | *Dicranomyia modesta* |
| 292 | Diptera | Limoniidae | *Dicranomyia sp.* |
| 293 | Diptera | Limoniidae | *Eloeophila maculata* |
| 294 | Diptera | Limoniidae | *Erioptera divisa* |
| 295 | Diptera | Limoniidae | *Erioptera sp.* |
| 296 | Diptera | Limoniidae | *Gonomyia tenella* |
| 297 | Diptera | Limoniidae | *Helius longirostris* |
| 298 | Diptera | Limoniidae | *Limonia nubeculosa* |
| 299 | Diptera | Limoniidae | *Limonia sp. BOLD:AAI1356* |
| 300 | Diptera | Limoniidae | *Limoniidae sp.* |
| 301 | Diptera | Limoniidae | *Metalimnobia bifasciata* |
| 302 | Diptera | Limoniidae | *Metalimnobia quadrinotata* |
| 303 | Diptera | Limoniidae | *Molophilus* |
| 304 | Diptera | Limoniidae | *Phylidorea ferruginea* |
| 305 | Diptera | Limoniidae | *Phylidorea squalens* |
| 306 | Diptera | Limoniidae | *Rhipidia maculata* |
| 307 | Diptera | Limoniidae | *Rhipidia sp.* |
| 308 | Diptera | Limoniidae | *Symplecta stictica* |
| 309 | Diptera | Muscidae | *Helina depuncta* |
| 310 | Diptera | Muscidae | *Helina evecta* |
| 311 | Diptera | Muscidae | *Helina impuncta* |
| 312 | Diptera | Muscidae | *Hydrotaea armipes* |
| 313 | Diptera | Muscidae | *Hydrotaea ignava* |
| 314 | Diptera | Muscidae | *Hydrotaea irritans* |
| 315 | Diptera | Muscidae | *Muscidae sp.* |
| 316 | Diptera | Muscidae | *Muscina levida* |
| 317 | Diptera | Muscidae | *Mydaea new sp. nr urbana* |
| 318 | Diptera | Muscidae | *Mydaea nr. nebulosa* |
| 319 | Diptera | Muscidae | *Phaonia sp.* |
| 320 | Diptera | Muscidae | *Polietes lardarius* |
| 321 | Diptera | Muscidae | *Thricops diaphanus* |
| 322 | Diptera | Muscidae | *Thricops rufisquamus* |
| 323 | Diptera | Mycetophilidae | *Coelosia fusca* |
| 324 | Diptera | Mycetophilidae | *Exechia seriata* |
| 325 | Diptera | Mycetophilidae | *Mycetophila luctuosa* |
| 326 | Diptera | Mycetophilidae | *Mycetophila sp.* |
| 327 | Diptera | Mycetophilidae | *Neuratelia nemoralis* |
| 328 | Diptera | Mycetophilidae | *Phronia sp.* |
| 329 | Diptera | Mycetophilidae | *Polylepta borealis* |
| 330 | Diptera | Mycetophilidae | *Sciophila krysheni* |
| 331 | Diptera | Mycetophilidae | *Sciophila lutea* |
| 332 | Diptera | Mycetophilidae | *Sciophila pseudoflexuosa* |
| 333 | Diptera | Pallopteridae | *Palloptera trimaculata* |
| 334 | Diptera | Pediciidae | *Dicranota gracilipes<ca>* |
| 335 | Diptera | Pediciidae | *Pedicia rivosa* |
| 336 | Diptera | Pediciidae | *Pediciidae sp.* |
| 337 | Diptera | Pediciidae | *Ula mixta* |
| 338 | Diptera | Pediciidae | *Ula sylvatica* |
| 339 | Diptera | Phoridae | *Megaselia arcticae* |
| 340 | Diptera | Phoridae | *Megaselia sp.* |
| 341 | Diptera | Psychodidae | *Pericoma blandula* |
| 342 | Diptera | Psychodidae | *Psychoda lativentris* |
| 343 | Diptera | Psychodidae | *Psychoda lobata* |
| 344 | Diptera | Psychodidae | *Psychoda sp.* |
| 345 | Diptera | Psychodidae | *Psychodidae sp.* |
| 346 | Diptera | Psychodidae | *Telmatoscopus advena* |
| 347 | Diptera | Rhagionidae | *Rhagio maculatus* |
| 348 | Diptera | Rhagionidae | *Rhagio scolopaceus* |
| 349 | Diptera | Rhagionidae | *Rhagio scolopaceus* |
| 350 | Diptera | Rhinophoridae | *Paykullia maculata* |
| 351 | Diptera | Sarcophagidae | *Sarcophaga pyrenaica* |
| 352 | Diptera | Scathophagidae | *Chaetosa punctipes* |
| 353 | Diptera | Scathophagidae | *Scathophaga suilla* |
| 354 | Diptera | Sciaridae | *Bradysia brevispina* |
| 355 | Diptera | Sciaridae | *Bradysia impatiens* |
| 356 | Diptera | Sciaridae | *Bradysia nitidicollis* |
| 357 | Diptera | Sciaridae | *Corynoptera boletiphaga* |
| 358 | Diptera | Sciaridae | *Cratyna nobilis* |
| 359 | Diptera | Sciaridae | *Ctenosciara hyalipennis* |
| 360 | Diptera | Sciaridae | *Leptosciarella saltuum* |
| 361 | Diptera | Sciaridae | *Lycoriella ingenua* |
| 362 | Diptera | Sciaridae | *Scatopsciara neglecta* |
| 363 | Diptera | Sciaridae | *Sciara hemerobioides* |
| 364 | Diptera | Sciaridae | *Sciaridae* |
| 365 | Diptera | Sciaridae | *Sciaridae sp.* |
| 366 | Diptera | Sciaridae | *Xylosciara betulae* |
| 367 | Diptera | Simuliidae | *Simulium equinum* |
| 368 | Diptera | Simuliidae | *Simulium erythrocephalum* |
| 369 | Diptera | Simuliidae | *Simulium intermedium* |
| 370 | Diptera | Simuliidae | *Simulium morsitans* |
| 371 | Diptera | Simuliidae | *Simulium noelleri* |
| 372 | Diptera | Simuliidae | *Simulium ornatum* |
| 373 | Diptera | Simuliidae | *Simulium pusillum* |
| 374 | Diptera | Simuliidae | *Simulium vernum* |
| 375 | Diptera | Stratiomyidae | *Beris chalybata* |
| 376 | Diptera | Syrphidae | *Meliscaeva cinctella* |
| 377 | Diptera | Syrphidae | *Parasyrphus annulatus* |
| 378 | Diptera | Syrphidae | *Syrphus ribesii* |
| 379 | Diptera | Syrphidae | *Syrphus torvus* |
| 380 | Diptera | Syrphidae | *Syrphus vitripennis* |
| 381 | Diptera | Syrphidae | *Xanthandrus comtus* |
| 382 | Diptera | Tabanidae | *Tabanus bromius* |
| 383 | Diptera | Tachinidae | *Bactromyia aurulenta* |
| 384 | Diptera | Tachinidae | *Ceromya silacea* |
| 385 | Diptera | Tachinidae | *Cyzenis albicans* |
| 386 | Diptera | Tachinidae | *Eloceria delecta* |
| 387 | Diptera | Tachinidae | *Gymnosoma nudifrons* |
| 388 | Diptera | Tachinidae | *Houghia graciloides* |
| 389 | Diptera | Tachinidae | *Loewia foeda* |
| 390 | Diptera | Tachinidae | *Lydina aenea* |
| 391 | Diptera | Tachinidae | *Lypha dubia* |
| 392 | Diptera | Tachinidae | *Macquartia dispar* |
| 393 | Diptera | Tachinidae | *Oswaldia muscaria* |
| 394 | Diptera | Tachinidae | *Pales pavida* |
| 395 | Diptera | Tachinidae | *Peleteria aenea* |
| 396 | Diptera | Tachinidae | *Peleteria setosa* |
| 397 | Diptera | Tachinidae | *Phebellia nigripalpis* |
| 398 | Diptera | Tachinidae | *Phorocera obscura* |
| 399 | Diptera | Tachinidae | *Siphona geniculata* |
| 400 | Diptera | Tachinidae | *Smidtia amoena* |
| 401 | Diptera | Tachinidae | *Smidtia fumiferanae* |
| 402 | Diptera | Tachinidae | *Tachinidae sp.* |
| 403 | Diptera | Tephritidae | *Dioxyna bidentis* |
| 404 | Diptera | Tephritidae | *Tephritidae sp.* |
| 405 | Diptera | Tipulidae | *Nephrotoma aculeata* |
| 406 | Diptera | Tipulidae | *Nephrotoma lunulicornis* |
| 407 | Diptera | Tipulidae | *Nigrotipula nigra* |
| 408 | Diptera | Tipulidae | *Tipula fascipennis* |
| 409 | Diptera | Tipulidae | *Tipula fulvipennis* |
| 410 | Diptera | Tipulidae | *Tipula lateralis* |
| 411 | Diptera | Tipulidae | *Tipula lunata* |
| 412 | Diptera | Tipulidae | *Tipula maxima* |
| 413 | Diptera | Tipulidae | *Tipula oleracea* |
| 414 | Diptera | Tipulidae | *Tipula paludosa* |
| 415 | Diptera | Tipulidae | *Tipula pierrei* |
| 416 | Diptera | Tipulidae | *Tipula scripta* |
| 417 | Diptera | Tipulidae | *Tipula sp.* |
| 418 | Diptera | Tipulidae | *Tipula sp. BOLD:AAG4539* |
| 419 | Diptera | Tipulidae | *Tipula sp. SOKN048* |
| 420 | Diptera | Tipulidae | *Tipula truncorum* |
| 421 | Diptera | Tipulidae | *Tipulidae sp.* |
| 422 | Diptera | Tipulidae | *Tipulidae sp. 4FA06* |
| 423 | Diptera | Tipulidae | *Tipulidae sp. BOLD:ACF0878* |
| 424 | Diptera | Trichoceridae | *Trichocera sp.* |
| 425 | Diptera | Trichoceridae | *Trichocera sp. BOLD:ACF7745* |
| 426 | Diptera | Xylophagidae | *Xylophagus ater* |
| 427 | Ephemeroptera | Baetidae | *Cloeon dipterum* |
| 428 | Ephemeroptera | Baetidae | *Procloeon bifidum* |
| 429 | Ephemeroptera | Caenidae | *Caenis horaria* |
| 430 | Ephemeroptera | Ephemeridae | *Ephemera vulgata* |
| 431 | Ephemeroptera | Heptageniidae | *Heptagenia sulphurea* |
| 432 | Ephemeroptera | Siphlonuridae | *Siphlonurus alternatus* |
| 433 | Hemiptera | Aphididae | *Anoecia* |
| 434 | Hemiptera | Aphididae | *Aphis craccae* |
| 435 | Hemiptera | Aphididae | *Aphis farinosa* |
| 436 | Hemiptera | Aphididae | *Aphis sp.* |
| 437 | Hemiptera | Aphididae | *Cinara piceicola* |
| 438 | Hemiptera | Aphididae | *Cinara pilosa* |
| 439 | Hemiptera | Aphididae | *Cinara pini* |
| 440 | Hemiptera | Aphididae | *Cinara sp. 3328* |
| 441 | Hemiptera | Aphididae | *Euceraphis betulae* |
| 442 | Hemiptera | Aphididae | *Euceraphis punctipennis* |
| 443 | Hemiptera | Aphididae | *Hyalopterus pruni* |
| 444 | Hemiptera | Aphididae | *Pachypappa rosettei* |
| 445 | Hemiptera | Aphididae | *Schizolachnus pineti* |
| 446 | Hemiptera | Aphididae | *Schizolachnus sp.* |
| 447 | Hemiptera | Cicadellidae | *Fagocyba douglasi* |
| 448 | Hemiptera | Corixidae | *Sigara falleni* |
| 449 | Hemiptera | Corixidae | *Sigara fossarum* |
| 450 | Hemiptera | Hemiptera | *Hemiptera sp.* |
| 451 | Hemiptera | Miridae | *Lygus borealis* |
| 452 | Hemiptera | Miridae | *Lygus pratensis* |
| 453 | Hemiptera | Miridae | *Miris striatus* |
| 454 | Hemiptera | Miridae | *Neolygus contaminatus* |
| 455 | Hemiptera | Miridae | *Phylus melanocephalus* |
| 456 | Hemiptera | Psyllidae | *Cacopsylla melanoneura* |
| 457 | Hemiptera | Psyllidae | *Psyllidae sp.* |
| 458 | Hymenoptera | Argidae | *Arge dimidiata* |
| 459 | Hymenoptera | Braconidae | *Braconidae sp.* |
| 460 | Hymenoptera | Braconidae | *Charmon sp. BF002040* |
| 461 | Hymenoptera | Braconidae | *Choeras jft30* |
| 462 | Hymenoptera | Braconidae | *Praon volucre* |
| 463 | Hymenoptera | Cimbicidae | *Cimbicidae sp.* |
| 464 | Hymenoptera | Hymenoptera | *Hymenoptera* |
| 465 | Hymenoptera | Ichneumonidae | *Agrypon flaveolatum* |
| 466 | Hymenoptera | Ichneumonidae | *Astiphromma splenium* |
| 467 | Hymenoptera | Ichneumonidae | *Diadegma fenestrale* |
| 468 | Hymenoptera | Ichneumonidae | *Diadegma majale* |
| 469 | Hymenoptera | Ichneumonidae | *Dusona insignita* |
| 470 | Hymenoptera | Ichneumonidae | *Eusterinx argutula* |
| 471 | Hymenoptera | Ichneumonidae | *Hyposoter PRO-3* |
| 472 | Hymenoptera | Ichneumonidae | *Ichneumonidae sp.* |
| 473 | Hymenoptera | Ichneumonidae | *Mesochorus sp.* |
| 474 | Hymenoptera | Ichneumonidae | *Mesochorus vitticollis* |
| 475 | Hymenoptera | Ichneumonidae | *Pleolophus sp.* |
| 476 | Hymenoptera | Ichneumonidae | *Sympherta obligator* |
| 477 | Hymenoptera | Tenthredinidae | *Ametastegia perla* |
| 478 | Hymenoptera | Tenthredinidae | *Dolerus vestigialis vestigialis* |
| 479 | Hymenoptera | Tenthredinidae | *Monophadnus pallescens* |
| 480 | Hymenoptera | Tenthredinidae | *Monsoma pulveratum* |
| 481 | Hymenoptera | Tenthredinidae | *Nematus dispar* |
| 482 | Hymenoptera | Tenthredinidae | *Pachyprotasis rapae* |
| 483 | Hymenoptera | Tenthredinidae | *Tenthredinidae sp.* |
| 484 | Lepidoptera | Adelidae | *Nematopogon magna* |
| 485 | Lepidoptera | Adelidae | *Nematopogon robertella* |
| 486 | Lepidoptera | Adelidae | *Nematopogon swammerdamellus* |
| 487 | Lepidoptera | Arctiidae | *Atolmis rubricollis* |
| 488 | Lepidoptera | Argyresthiidae | *Argyresthia abdominalis* |
| 489 | Lepidoptera | Argyresthiidae | *Argyresthia bergiella* |
| 490 | Lepidoptera | Argyresthiidae | *Argyresthia goedartella* |
| 491 | Lepidoptera | Argyresthiidae | *Argyresthia retinella* |
| 492 | Lepidoptera | Batrachedridae | *Batrachedra pinicolella* |
| 493 | Lepidoptera | Batrachedridae | *Batrachedra praeangusta* |
| 494 | Lepidoptera | Bombycidae | *Bombyx mori* |
| 495 | Lepidoptera | Bucculatricidae | *Bucculatrix cidarella* |
| 496 | Lepidoptera | Bucculatricidae | *Bucculatrix thoracella* |
| 497 | Lepidoptera | Bucculatricidae | *Bucculatrix ulmella* |
| 498 | Lepidoptera | Coleophoridae | *Coleophora alcyonipennella* |
| 499 | Lepidoptera | Coleophoridae | *Coleophora alticolella* |
| 500 | Lepidoptera | Coleophoridae | *Coleophora betulella* |
| 501 | Lepidoptera | Coleophoridae | *Coleophora flavipennella* |
| 502 | Lepidoptera | Coleophoridae | *Coleophora kuehnella* |
| 503 | Lepidoptera | Coleophoridae | *Coleophora sp.* |
| 504 | Lepidoptera | Coleophoridae | *Coleophora spinella* |
| 505 | Lepidoptera | Coleophoridae | *Coleophora versurella* |
| 506 | Lepidoptera | Coleophoridae | *Coleophoridae sp.* |
| 507 | Lepidoptera | Cosmopterigidae | *Limnaecia phragmitella* |
| 508 | Lepidoptera | Cosmopterigidae | *Sorhagenia janiszewskae* |
| 509 | Lepidoptera | Crambidae | *Acentria ephemerella* |
| 510 | Lepidoptera | Crambidae | *Agriphila inquinatella* |
| 511 | Lepidoptera | Crambidae | *Agriphila selasella* |
| 512 | Lepidoptera | Crambidae | *Agriphila straminella* |
| 513 | Lepidoptera | Crambidae | *Calamotropha paludella* |
| 514 | Lepidoptera | Crambidae | *Chrysoteuchia culmella* |
| 515 | Lepidoptera | Crambidae | *Crambus lathoniellus* |
| 516 | Lepidoptera | Crambidae | *Crambus pascuellus* |
| 517 | Lepidoptera | Crambidae | *Donacaula mucronella* |
| 518 | Lepidoptera | Crambidae | *Donacaula sp.* |
| 519 | Lepidoptera | Crambidae | *Elophila nymphaeata* |
| 520 | Lepidoptera | Crambidae | *Evergestis extimalis* |
| 521 | Lepidoptera | Crambidae | *Nacoleia commixta* |
| 522 | Lepidoptera | Crambidae | *Nymphula nitidulata* |
| 523 | Lepidoptera | Crambidae | *Ostrinia nubilalis* |
| 524 | Lepidoptera | Crambidae | *Palpita quadristigmalis* |
| 525 | Lepidoptera | Crambidae | *Preneopogon catenalis* |
| 526 | Lepidoptera | Crambidae | *Scoparia ancipitella* |
| 527 | Lepidoptera | Crambidae | *Scoparia subfusca* |
| 528 | Lepidoptera | Crambidae | *Udea lutealis* |
| 529 | Lepidoptera | Depressariidae | *Agonopterix angelicella* |
| 530 | Lepidoptera | Depressariidae | *Agonopterix arenella* |
| 531 | Lepidoptera | Depressariidae | *Agonopterix ciliella* |
| 532 | Lepidoptera | Depressariidae | *Agonopterix heracliana* |
| 533 | Lepidoptera | Depressariidae | *Agonopterix multiplicella* |
| 534 | Lepidoptera | Depressariidae | *Agonopterix ocellana* |
| 535 | Lepidoptera | Depressariidae | *Agonopterix propinquella* |
| 536 | Lepidoptera | Depressariidae | *Depressaria daucella* |
| 537 | Lepidoptera | Depressariidae | *Depressaria emeritella* |
| 538 | Lepidoptera | Depressariidae | *Depressaria libanotidella* |
| 539 | Lepidoptera | Depressariidae | *Depressaria olerella* |
| 540 | Lepidoptera | Depressariidae | *Depressaria radiella* |
| 541 | Lepidoptera | Depressariidae | *Depressaria sordidatella* |
| 542 | Lepidoptera | Drepanidae | *Achlya flavicornis* |
| 543 | Lepidoptera | Drepanidae | *Drepana falcataria* |
| 544 | Lepidoptera | Drepanidae | *Falcaria lacertinaria* |
| 545 | Lepidoptera | Drepanidae | *Tethea or* |
| 546 | Lepidoptera | Drepanidae | *Tetheella fluctuosa* |
| 547 | Lepidoptera | Elachistidae | *Elachista adscitella* |
| 548 | Lepidoptera | Elachistidae | *Elachista subalbidella* |
| 549 | Lepidoptera | Endromidae | *Endromis versicolora* |
| 550 | Lepidoptera | Epermeniidae | *Epermenia illigerella* |
| 551 | Lepidoptera | Erebidae | *Calliteara pudibunda* |
| 552 | Lepidoptera | Erebidae | *Catocala lara* |
| 553 | Lepidoptera | Erebidae | *Dasychira pseudabietis* |
| 554 | Lepidoptera | Erebidae | *Diacrisia sannio* |
| 555 | Lepidoptera | Erebidae | *Eilema depressum* |
| 556 | Lepidoptera | Erebidae | *Erebidae sp.* |
| 557 | Lepidoptera | Erebidae | *Herminia tarsipennalis* |
| 558 | Lepidoptera | Erebidae | *Hypena crassalis* |
| 559 | Lepidoptera | Erebidae | *Hypena proboscidalis* |
| 560 | Lepidoptera | Erebidae | *Lygephila pastinum* |
| 561 | Lepidoptera | Erebidae | *Lymantria monacha* |
| 562 | Lepidoptera | Erebidae | *Lymantria umbrosa* |
| 563 | Lepidoptera | Erebidae | *Macrochilo cribrumalis* |
| 564 | Lepidoptera | Erebidae | *Rivula sericealis* |
| 565 | Lepidoptera | Erebidae | *Scoliopteryx libatrix* |
| 566 | Lepidoptera | Erebidae | *Spilarctia luteum* |
| 567 | Lepidoptera | Gelechiidae | *Acompsia cinerella* |
| 568 | Lepidoptera | Gelechiidae | *Carpatolechia fugitivella* |
| 569 | Lepidoptera | Gelechiidae | *Carpatolechia proximella* |
| 570 | Lepidoptera | Gelechiidae | *Caryocolum pullatella* |
| 571 | Lepidoptera | Gelechiidae | *Caryocolum vicinella* |
| 572 | Lepidoptera | Gelechiidae | *Chionodes electella* |
| 573 | Lepidoptera | Gelechiidae | *Chionodes lugubrella* |
| 574 | Lepidoptera | Gelechiidae | *Dichomeris alacella* |
| 575 | Lepidoptera | Gelechiidae | *Exoteleia dodecella* |
| 576 | Lepidoptera | Gelechiidae | *Gelechia cuneatella* |
| 577 | Lepidoptera | Gelechiidae | *Gelechia muscosella* |
| 578 | Lepidoptera | Gelechiidae | *Gelechia nigra* |
| 579 | Lepidoptera | Gelechiidae | *Gelechia sabinella* |
| 580 | Lepidoptera | Gelechiidae | *Gelechia sororculella* |
| 581 | Lepidoptera | Gelechiidae | *Helcystogramma rufescens* |
| 582 | Lepidoptera | Gelechiidae | *Monochroa conspersella* |
| 583 | Lepidoptera | Gelechiidae | *Monochroa lutulentella* |
| 584 | Lepidoptera | Gelechiidae | *Neofriseria peliella* |
| 585 | Lepidoptera | Gelechiidae | *Neofriseria sp.* |
| 586 | Lepidoptera | Gelechiidae | *Psoricoptera gibbosella* |
| 587 | Lepidoptera | Gelechiidae | *Recurvaria leucatella* |
| 588 | Lepidoptera | Gelechiidae | *Scrobipalpa atriplicella* |
| 589 | Lepidoptera | Gelechiidae | *Teleiopsis diffinis* |
| 590 | Lepidoptera | Geometridae | *Aethalura punctulata* |
| 591 | Lepidoptera | Geometridae | *Agriopis aurantiaria* |
| 592 | Lepidoptera | Geometridae | *Alcis repandata* |
| 593 | Lepidoptera | Geometridae | *Apocheima pilosaria* |
| 594 | Lepidoptera | Geometridae | *Biston stratarius* |
| 595 | Lepidoptera | Geometridae | *Bupalus piniaria* |
| 596 | Lepidoptera | Geometridae | *Cabera exanthemata* |
| 597 | Lepidoptera | Geometridae | *Cabera pusaria* |
| 598 | Lepidoptera | Geometridae | *Cleora cinctaria* |
| 599 | Lepidoptera | Geometridae | *Colostygia olivata* |
| 600 | Lepidoptera | Geometridae | *Colotois pennaria* |
| 601 | Lepidoptera | Geometridae | *Crocallis elinguaria* |
| 602 | Lepidoptera | Geometridae | *Deileptenia ribeata* |
| 603 | Lepidoptera | Geometridae | *Ectropis crepuscularia* |
| 604 | Lepidoptera | Geometridae | *Epirrhoe alternata* |
| 605 | Lepidoptera | Geometridae | *Epirrita autumnata* |
| 606 | Lepidoptera | Geometridae | *Erannis defoliaria* |
| 607 | Lepidoptera | Geometridae | *Eulithis populata* |
| 608 | Lepidoptera | Geometridae | *Eulithis testata* |
| 609 | Lepidoptera | Geometridae | *Eupithecia abietaria* |
| 610 | Lepidoptera | Geometridae | *Eupithecia indigata* |
| 611 | Lepidoptera | Geometridae | *Eupithecia lanceata* |
| 612 | Lepidoptera | Geometridae | *Eupithecia plumbeolata* |
| 613 | Lepidoptera | Geometridae | *Eupithecia sp.* |
| 614 | Lepidoptera | Geometridae | *Eupithecia subfuscata* |
| 615 | Lepidoptera | Geometridae | *Eupithecia tantillaria* |
| 616 | Lepidoptera | Geometridae | *Eupithecia tenuiata* |
| 617 | Lepidoptera | Geometridae | *Eupithecia virgaureata* |
| 618 | Lepidoptera | Geometridae | *Gandaritis pyraliata* |
| 619 | Lepidoptera | Geometridae | *Geometridae sp.* |
| 620 | Lepidoptera | Geometridae | *Gnophos obfuscata* |
| 621 | Lepidoptera | Geometridae | *Gymnoscelis rufifasciata* |
| 622 | Lepidoptera | Geometridae | *Hypomecis punctinalis* |
| 623 | Lepidoptera | Geometridae | *Idaea biselata* |
| 624 | Lepidoptera | Geometridae | *Idaea dimidiata* |
| 625 | Lepidoptera | Geometridae | *Idaea emarginata* |
| 626 | Lepidoptera | Geometridae | *Lobophora halterata* |
| 627 | Lepidoptera | Geometridae | *Lomaspilis marginata* |
| 628 | Lepidoptera | Geometridae | *Macaria brunneata* |
| 629 | Lepidoptera | Geometridae | *Macaria liturata* |
| 630 | Lepidoptera | Geometridae | *Odontopera bidentata* |
| 631 | Lepidoptera | Geometridae | *Operophtera brumata* |
| 632 | Lepidoptera | Geometridae | *Operophtera brumata x O. bruceata* |
| 633 | Lepidoptera | Geometridae | *Paradarisa consonaria* |
| 634 | Lepidoptera | Geometridae | *Pasiphila rectangulata* |
| 635 | Lepidoptera | Geometridae | *Plagodis pulveraria* |
| 636 | Lepidoptera | Geometridae | *Rheumaptera undulata* |
| 637 | Lepidoptera | Geometridae | *Scopula floslactata* |
| 638 | Lepidoptera | Geometridae | *Scopula immutata* |
| 639 | Lepidoptera | Geometridae | *Scotopteryx chenopodiata* |
| 640 | Lepidoptera | Geometridae | *Selenia dentaria* |
| 641 | Lepidoptera | Geometridae | *Xanthorhoe fluctuata* |
| 642 | Lepidoptera | Geometridae | *Xanthorhoe montanata* |
| 643 | Lepidoptera | Geometridae | *Xanthorhoe quadrifasciata* |
| 644 | Lepidoptera | Geometridae | *Xanthorhoe spadicearia* |
| 645 | Lepidoptera | Glyphipterigidae | *Glyphipterix simpliciella* |
| 646 | Lepidoptera | Glyphipterigidae | *Orthotelia sparganella* |
| 647 | Lepidoptera | Gracillariidae | *Caloptilia alchimiella* |
| 648 | Lepidoptera | Gracillariidae | *Caloptilia betulicola* |
| 649 | Lepidoptera | Gracillariidae | *Caloptilia elongella* |
| 650 | Lepidoptera | Gracillariidae | *Caloptilia hemidactylella* |
| 651 | Lepidoptera | Gracillariidae | *Caloptilia populetorum* |
| 652 | Lepidoptera | Gracillariidae | *Parornix betulae* |
| 653 | Lepidoptera | Gracillariidae | *Parornix devoniella* |
| 654 | Lepidoptera | Gracillariidae | *Phyllonorycter apparella* |
| 655 | Lepidoptera | Gracillariidae | *Phyllonorycter harrisella* |
| 656 | Lepidoptera | Gracillariidae | *Phyllonorycter nigrescentella* |
| 657 | Lepidoptera | Hepialidae | *Pharmacis fusconebulosa* |
| 658 | Lepidoptera | Hesperiidae | *Callophrys irus* |
| 659 | Lepidoptera | Hesperiidae | *Pyrgus centaureae* |
| 660 | Lepidoptera | Hesperiidae | *Staphylus evemerus* |
| 661 | Lepidoptera | Lasiocampidae | *Dendrolimus pini* |
| 662 | Lepidoptera | Lasiocampidae | *Lasiocampa quercus* |
| 663 | Lepidoptera | Lasiocampidae | *Macrothylacia rubi* |
| 664 | Lepidoptera | Lepidoptera fam. | *Lepidoptera sp.* |
| 665 | Lepidoptera | Lycaenidae | *Lycaenidae sp.* |
| 666 | Lepidoptera | Lyonetiidae | *Lyonetia clerkella* |
| 667 | Lepidoptera | Momphidae | *Mompha sturnipennella* |
| 668 | Lepidoptera | Momphidae | *Mompha subbistrigella* |
| 669 | Lepidoptera | Noctuidae | *Acronicta auricoma* |
| 670 | Lepidoptera | Noctuidae | *Acronicta rumicis* |
| 671 | Lepidoptera | Noctuidae | *Agrochola helvola* |
| 672 | Lepidoptera | Noctuidae | *Agrotis clavis* |
| 673 | Lepidoptera | Noctuidae | *Agrotis exclamationis* |
| 674 | Lepidoptera | Noctuidae | *Allophyes oxyacanthae* |
| 675 | Lepidoptera | Noctuidae | *Amphipyra berbera* |
| 676 | Lepidoptera | Noctuidae | *Amphipyra pyramidea* |
| 677 | Lepidoptera | Noctuidae | *Apamea crenata* |
| 678 | Lepidoptera | Noctuidae | *Apamea remissa* |
| 679 | Lepidoptera | Noctuidae | *Apamea scolopacina* |
| 680 | Lepidoptera | Noctuidae | *Apamea sordens* |
| 681 | Lepidoptera | Noctuidae | *Autographa gamma* |
| 682 | Lepidoptera | Noctuidae | *Autographa pulchrina* |
| 683 | Lepidoptera | Noctuidae | *Brachionycha nubeculosa* |
| 684 | Lepidoptera | Noctuidae | *Caradrina morpheus* |
| 685 | Lepidoptera | Noctuidae | *Cerastis rubricosa* |
| 686 | Lepidoptera | Noctuidae | *Charadra deridens* |
| 687 | Lepidoptera | Noctuidae | *Charanyca ferruginea* |
| 688 | Lepidoptera | Noctuidae | *Chloantha hyperici* |
| 689 | Lepidoptera | Noctuidae | *Colocasia coryli* |
| 690 | Lepidoptera | Noctuidae | *Conistra rubiginea* |
| 691 | Lepidoptera | Noctuidae | *Conistra vaccinii* |
| 692 | Lepidoptera | Noctuidae | *Cosmia trapezina* |
| 693 | Lepidoptera | Noctuidae | *Diarsia rubi* |
| 694 | Lepidoptera | Noctuidae | *Eurois occultus* |
| 695 | Lepidoptera | Noctuidae | *Hada plebeja* |
| 696 | Lepidoptera | Noctuidae | *Helotropha leucostigma* |
| 697 | Lepidoptera | Noctuidae | *Hillia iris* |
| 698 | Lepidoptera | Noctuidae | *Hoplodrina octogenaria* |
| 699 | Lepidoptera | Noctuidae | *Hydraecia micacea* |
| 700 | Lepidoptera | Noctuidae | *Hyppa rectilinea* |
| 701 | Lepidoptera | Noctuidae | *Lenisa geminipuncta* |
| 702 | Lepidoptera | Noctuidae | *Lithophane furcifera* |
| 703 | Lepidoptera | Noctuidae | *Lithophane socia* |
| 704 | Lepidoptera | Noctuidae | *Mesapamea secalis* |
| 705 | Lepidoptera | Noctuidae | *Mniotype bathensis* |
| 706 | Lepidoptera | Noctuidae | *Naenia typica* |
| 707 | Lepidoptera | Noctuidae | *Noctuidae sp.* |
| 708 | Lepidoptera | Noctuidae | *Nonagria typhae* |
| 709 | Lepidoptera | Noctuidae | *Oligia latruncula* |
| 710 | Lepidoptera | Noctuidae | *Orthosia gothica* |
| 711 | Lepidoptera | Noctuidae | *Orthosia opima* |
| 712 | Lepidoptera | Noctuidae | *Panolis flammea* |
| 713 | Lepidoptera | Noctuidae | *Panthea coenobita* |
| 714 | Lepidoptera | Noctuidae | *Polia bombycina* |
| 715 | Lepidoptera | Noctuidae | *Polia hepatica* |
| 716 | Lepidoptera | Noctuidae | *Protolampra sobrina* |
| 717 | Lepidoptera | Noctuidae | *Protoschinia scutosa* |
| 718 | Lepidoptera | Noctuidae | *Sideridis reticulata* |
| 719 | Lepidoptera | Noctuidae | *Subacronicta megacephala* |
| 720 | Lepidoptera | Noctuidae | *Sympistis zetterstedtii* |
| 721 | Lepidoptera | Noctuidae | *Tholera decimalis* |
| 722 | Lepidoptera | Noctuidae | *Trachea atriplicis* |
| 723 | Lepidoptera | Noctuidae | *Xestia baja* |
| 724 | Lepidoptera | Noctuidae | *Xestia triangulum* |
| 725 | Lepidoptera | Noctuidae | *Xylena vetusta* |
| 726 | Lepidoptera | Nolidae | *Nycteola degenerana* |
| 727 | Lepidoptera | Nolidae | *Nycteola revayana* |
| 728 | Lepidoptera | Notodontidae | *Cerura vinula* |
| 729 | Lepidoptera | Notodontidae | *Notodonta dromedarius* |
| 730 | Lepidoptera | Notodontidae | *Pheosia gnoma* |
| 731 | Lepidoptera | Notodontidae | *Ptilodon capucinus* |
| 732 | Lepidoptera | Nymphalidae | *Argynnis paphia* |
| 733 | Lepidoptera | Nymphalidae | *Boloria chariclea* |
| 734 | Lepidoptera | Oecophoridae | *Crassa tinctella* |
| 735 | Lepidoptera | Oecophoridae | *Denisia obscurella* |
| 736 | Lepidoptera | Oecophoridae | *Denisia stipella* |
| 737 | Lepidoptera | Oecophoridae | *Pseudatemelia elsae* |
| 738 | Lepidoptera | Oecophoridae | *Pseudatemelia josephinae* |
| 739 | Lepidoptera | Pieridae | *Colias palaeno* |
| 740 | Lepidoptera | Plutellidae | *Plutella xylostella* |
| 741 | Lepidoptera | Praydidae | *Prays fraxinella* |
| 742 | Lepidoptera | Psychidae | *Taleporia borealis* |
| 743 | Lepidoptera | Pterophoridae | *Gillmeria pallidactyla* |
| 744 | Lepidoptera | Pyralidae | *Dioryctria abietella* |
| 745 | Lepidoptera | Pyralidae | *Hypochalcia ahenella* |
| 746 | Lepidoptera | Pyralidae | *Phycita roborella* |
| 747 | Lepidoptera | Saturniidae | *Aglia tau* |
| 748 | Lepidoptera | Saturniidae | *Saturnia pavonia* |
| 749 | Lepidoptera | Saturniidae | *Saturniidae sp.* |
| 750 | Lepidoptera | Sphingidae | *Deilephila elpenor* |
| 751 | Lepidoptera | Sphingidae | *Laothoe populi* |
| 752 | Lepidoptera | Tineidae | *Archinemapogon yildizae* |
| 753 | Lepidoptera | Tineidae | *Infurcitinea sp.* |
| 754 | Lepidoptera | Tineidae | *Montescardia tessulatellus* |
| 755 | Lepidoptera | Tineidae | *Morophaga choragella* |
| 756 | Lepidoptera | Tineidae | *Nemapogon cloacella* |
| 757 | Lepidoptera | Tineidae | *Nemapogon nigralbella* |
| 758 | Lepidoptera | Tineidae | *Nemaxera betulinella* |
| 759 | Lepidoptera | Tineidae | *Niditinea striolella* |
| 760 | Lepidoptera | Tineidae | *Triaxomera fulvimitrella* |
| 761 | Lepidoptera | Tischeriidae | *Tischeria dodonaea* |
| 762 | Lepidoptera | Tischeriidae | *Tischeria ekebladella* |
| 763 | Lepidoptera | Tortricidae | *Acleris aspersana* |
| 764 | Lepidoptera | Tortricidae | *Acleris forsskaleana* |
| 765 | Lepidoptera | Tortricidae | *Acleris hastiana* |
| 766 | Lepidoptera | Tortricidae | *Acleris holmiana* |
| 767 | Lepidoptera | Tortricidae | *Acleris laterana* |
| 768 | Lepidoptera | Tortricidae | *Acleris lipsiana* |
| 769 | Lepidoptera | Tortricidae | *Acleris logiana* |
| 770 | Lepidoptera | Tortricidae | *Acleris notana* |
| 771 | Lepidoptera | Tortricidae | *Acleris rhombana* |
| 772 | Lepidoptera | Tortricidae | *Acleris sp.* |
| 773 | Lepidoptera | Tortricidae | *Adoxophyes orana* |
| 774 | Lepidoptera | Tortricidae | *Aethes smeathmanniana* |
| 775 | Lepidoptera | Tortricidae | *Agapeta hamana* |
| 776 | Lepidoptera | Tortricidae | *Aleimma loeflingiana* |
| 777 | Lepidoptera | Tortricidae | *Ancylis badiana* |
| 778 | Lepidoptera | Tortricidae | *Ancylis laetana* |
| 779 | Lepidoptera | Tortricidae | *Ancylis mitterbacheriana* |
| 780 | Lepidoptera | Tortricidae | *Ancylis myrtillana* |
| 781 | Lepidoptera | Tortricidae | *Aphelia paleana* |
| 782 | Lepidoptera | Tortricidae | *Apotomis fraterculana* |
| 783 | Lepidoptera | Tortricidae | *Apotomis infida* |
| 784 | Lepidoptera | Tortricidae | *Apotomis inundana* |
| 785 | Lepidoptera | Tortricidae | *Apotomis sauciana* |
| 786 | Lepidoptera | Tortricidae | *Archips oporana* |
| 787 | Lepidoptera | Tortricidae | *Archips podana* |
| 788 | Lepidoptera | Tortricidae | *Archips podanus* |
| 789 | Lepidoptera | Tortricidae | *Archips xylosteana* |
| 790 | Lepidoptera | Tortricidae | *Bactra furfurana* |
| 791 | Lepidoptera | Tortricidae | *Bactra lancealana* |
| 792 | Lepidoptera | Tortricidae | *Celypha rivulana* |
| 793 | Lepidoptera | Tortricidae | *Celypha striana* |
| 794 | Lepidoptera | Tortricidae | *Clepsis senecionana* |
| 795 | Lepidoptera | Tortricidae | *Clepsis spectrana* |
| 796 | Lepidoptera | Tortricidae | *Cnephasia asseclana* |
| 797 | Lepidoptera | Tortricidae | *Cnephasia stephensiana* |
| 798 | Lepidoptera | Tortricidae | *Cochylis nana* |
| 799 | Lepidoptera | Tortricidae | *Eana argentana* |
| 800 | Lepidoptera | Tortricidae | *Eana incanana* |
| 801 | Lepidoptera | Tortricidae | *Enarmonia formosana* |
| 802 | Lepidoptera | Tortricidae | *Epiblema scutulana* |
| 803 | Lepidoptera | Tortricidae | *Epiblema sticticana* |
| 804 | Lepidoptera | Tortricidae | *Epinotia bilunana* |
| 805 | Lepidoptera | Tortricidae | *Epinotia cinereana* |
| 806 | Lepidoptera | Tortricidae | *Epinotia nanana* |
| 807 | Lepidoptera | Tortricidae | *Epinotia nisella* |
| 808 | Lepidoptera | Tortricidae | *Epinotia signatana* |
| 809 | Lepidoptera | Tortricidae | *Epinotia solandriana* |
| 810 | Lepidoptera | Tortricidae | *Epinotia tedella* |
| 811 | Lepidoptera | Tortricidae | *Epinotia tenerana* |
| 812 | Lepidoptera | Tortricidae | *Epinotia tetraquetrana* |
| 813 | Lepidoptera | Tortricidae | *Eucosma cana* |
| 814 | Lepidoptera | Tortricidae | *Eucosma hohenwartiana* |
| 815 | Lepidoptera | Tortricidae | *Eudemis porphyrana* |
| 816 | Lepidoptera | Tortricidae | *Grapholita compositella* |
| 817 | Lepidoptera | Tortricidae | *Gypsonoma dealbana* |
| 818 | Lepidoptera | Tortricidae | *Hedya nubiferana* |
| 819 | Lepidoptera | Tortricidae | *Hedya ochroleucana* |
| 820 | Lepidoptera | Tortricidae | *Lathronympha strigana* |
| 821 | Lepidoptera | Tortricidae | *Lobesia reliquana* |
| 822 | Lepidoptera | Tortricidae | *Metendothenia atropunctana* |
| 823 | Lepidoptera | Tortricidae | *Notocelia cynosbatella* |
| 824 | Lepidoptera | Tortricidae | *Orthotaenia undulana* |
| 825 | Lepidoptera | Tortricidae | *Pandemis cerasana* |
| 826 | Lepidoptera | Tortricidae | *Pandemis cinnamomeana* |
| 827 | Lepidoptera | Tortricidae | *Paramesia gnomana* |
| 828 | Lepidoptera | Tortricidae | *Phalonidia udana* |
| 829 | Lepidoptera | Tortricidae | *Piniphila bifasciana* |
| 830 | Lepidoptera | Tortricidae | *Pseudargyrotoza conwagana* |
| 831 | Lepidoptera | Tortricidae | *Ptycholoma lecheana* |
| 832 | Lepidoptera | Tortricidae | *Rhopobota naevana* |
| 833 | Lepidoptera | Tortricidae | *Rhyacionia buoliana* |
| 834 | Lepidoptera | Tortricidae | *Spilonota eremitana* |
| 835 | Lepidoptera | Tortricidae | *Spilonota ocellana* |
| 836 | Lepidoptera | Tortricidae | *Syndemis musculana* |
| 837 | Lepidoptera | Tortricidae | *Thiodia citrana* |
| 838 | Lepidoptera | Tortricidae | *Tortrix viridana* |
| 839 | Lepidoptera | Tortricidae | *Zeiraphera isertana* |
| 840 | Lepidoptera | Tortricidae | *Zeiraphera ratzeburgiana* |
| 841 | Lepidoptera | Yponomeutidae | *Argyresthia arceuthina* |
| 842 | Lepidoptera | Yponomeutidae | *Argyresthia brockeella* |
| 843 | Lepidoptera | Yponomeutidae | *Argyresthia conjugella* |
| 844 | Lepidoptera | Yponomeutidae | *Argyresthia glabratella* |
| 845 | Lepidoptera | Yponomeutidae | *Argyresthia sp.* |
| 846 | Lepidoptera | Yponomeutidae | *Cedestis gysseleniella* |
| 847 | Lepidoptera | Yponomeutidae | *Cedestis subfasciella* |
| 848 | Lepidoptera | Yponomeutidae | *Paraswammerdamia conspersella* |
| 849 | Lepidoptera | Yponomeutidae | *Paraswammerdamia nebulella* |
| 850 | Lepidoptera | Ypsolophidae | *Ypsolopha asperella* |
| 851 | Lepidoptera | Ypsolophidae | *Ypsolopha falcella* |
| 852 | Lepidoptera | Ypsolophidae | *Ypsolopha parenthesella* |
| 853 | Lepidoptera | Ypsolophidae | *Ypsolopha scabrella* |
| 854 | Lepidoptera | Ypsolophidae | *Ypsolopha sp.* |
| 855 | Lepidoptera | Ypsolophidae | *Ypsolopha sylvella* |
| 856 | Lepidoptera | Ypsolophidae | *Ypsolopha ustella* |
| 857 | Megaloptera | Sialidae | *Sialis lutaria* |
| 858 | Neuroptera | Chrysopidae | *Chrysopa pallens* |
| 859 | Neuroptera | Chrysopidae | *Chrysoperla carnea* |
| 860 | Neuroptera | Chrysopidae | *Chrysoperla pallida* |
| 861 | Neuroptera | Chrysopidae | *Cunctochrysa albolineata* |
| 862 | Neuroptera | Hemerobiidae | *Hemerobius contumax* |
| 863 | Neuroptera | Hemerobiidae | *Hemerobius fenestratus* |
| 864 | Neuroptera | Hemerobiidae | *Hemerobius humulinus* |
| 865 | Neuroptera | Hemerobiidae | *Hemerobius pini* |
| 866 | Neuroptera | Hemerobiidae | *Hemerobius simulans* |
| 867 | Neuroptera | Hemerobiidae | *Hemerobius stigma* |
| 868 | Neuroptera | Hemerobiidae | *Wesmaelius concinnus* |
| 869 | Neuroptera | Neuroptera fam. | *Neuroptera sp.* |
| 870 | Neuroptera | Sisyridae | *Sisyra nigra* |
| 871 | Orthoptera | Gryllidae | *Gryllus bimaculatus* |
| 872 | Orthoptera | Tettigoniidae | *Pholidoptera griseoaptera* |
| 873 | Plecoptera | Leuctridae | *Leuctra digitata* |
| 874 | Plecoptera | Leuctridae | *Leuctra fusca* |
| 875 | Plecoptera | Nemouridae | *Nemoura cinerea* |
| 876 | Psocodea | Caeciliusidae | *Valenzuela flavidus* |
| 877 | Psocodea | Lachesillidae | *Lachesilla pedicularia* |
| 878 | Psocodea | Peripsocidae | *Peripsocus subfasciatus* |
| 879 | Psocodea | Psocidae | *Loensia fasciata* |
| 880 | Psocodea | Psocodea fam. | *Psocodea sp.* |
| 881 | Trichoptera | Goeridae | *Goera pilosa* |
| 882 | Trichoptera | Lepidostomatidae | *Lepidostoma hirtum* |
| 883 | Trichoptera | Leptoceridae | *Athripsodes cinereus* |
| 884 | Trichoptera | Leptoceridae | *Ceraclea albimacula* |
| 885 | Trichoptera | Leptoceridae | *Ceraclea annulicornis* |
| 886 | Trichoptera | Leptoceridae | *Ceraclea dissimilis* |
| 887 | Trichoptera | Leptoceridae | *Ceraclea excisa* |
| 888 | Trichoptera | Leptoceridae | *Ceraclea fulva* |
| 889 | Trichoptera | Leptoceridae | *Ceraclea senilis* |
| 890 | Trichoptera | Leptoceridae | *Ceraclea sp.* |
| 891 | Trichoptera | Leptoceridae | *Mystacides azureus* |
| 892 | Trichoptera | Leptoceridae | *Mystacides longicornis* |
| 893 | Trichoptera | Leptoceridae | *Mystacides nigra* |
| 894 | Trichoptera | Leptoceridae | *Oecetis furva* |
| 895 | Trichoptera | Leptoceridae | *Oecetis lacustris* |
| 896 | Trichoptera | Leptoceridae | *Oecetis ochracea* |
| 897 | Trichoptera | Leptoceridae | *Oecetis testacea* |
| 898 | Trichoptera | Leptoceridae | *Triaenodes bicolor* |
| 899 | Trichoptera | Leptoceridae | *Triaenodes detruncatus* |
| 900 | Trichoptera | Limnephilidae | *Glyphotaelius pellucidus* |
| 901 | Trichoptera | Limnephilidae | *Halesus digitatus* |
| 902 | Trichoptera | Limnephilidae | *Halesus tessellatus* |
| 903 | Trichoptera | Limnephilidae | *Limnephilus affinis* |
| 904 | Trichoptera | Limnephilidae | *Limnephilus flavicornis* |
| 905 | Trichoptera | Limnephilidae | *Limnephilus fuscicornis* |
| 906 | Trichoptera | Limnephilidae | *Limnephilus ignavus* |
| 907 | Trichoptera | Limnephilidae | *Limnephilus sparsus* |
| 908 | Trichoptera | Limnephilidae | *Micropterna sequax* |
| 909 | Trichoptera | Limnephilidae | *Rhadicoleptus alpestris* |
| 910 | Trichoptera | Limnephilidae | *Stenophylax lateralis* |
| 911 | Trichoptera | Limnephilidae | *Stenophylax sequax* |
| 912 | Trichoptera | Molannidae | *Molanna angustata* |
| 913 | Trichoptera | Phryganeidae | *Agrypnia obsoleta* |
| 914 | Trichoptera | Phryganeidae | *Agrypnia pagetana hyperborea* |
| 915 | Trichoptera | Phryganeidae | *Agrypnia varia* |
| 916 | Trichoptera | Phryganeidae | *Phryganea bipunctata* |
| 917 | Trichoptera | Phryganeidae | *Phryganea grandis* |
| 918 | Trichoptera | Polycentropodidae | *Cyrnus trimaculatus* |
| 919 | Trichoptera | Polycentropodidae | *Plectrocnemia conspersa* |
| 920 | Trichoptera | Polycentropodidae | *Polycentropus flavomaculatus* |
| 921 | Trichoptera | Psychomyiidae | *Lype phaeopa* |
| 922 | Trichoptera | Psychomyiidae | *Psychomyia pusilla* |
| 923 | Trichoptera | Rhyacophilidae | *Rhyacophila nubila* |
|  | MALACOSTRACA |  |  |
| 924 | Isopoda | Asellidae | *Asellus aquaticus* |

Figure S2. The prey web species numbers which correspond to the prey species list Table S1.

Figure S3. Prey family level web including names for each family.

Figure S4. Prey order level web including names for each order.

### Species accumulation curves

We calculated species accumulation curves for each predator based on read counts (Figure S5) and number of samples (Figure S6). *Myotis mystacinus* was represented by only one pooled sample, and no accumulation curve was calculated.

Figure S5. Species accumulation curves for each sampled predator, based on prey reads (x-axis) and detected number of prey species (y-axis). The lowest row is based on a data from a pilot study, with moderate sampling effort and low number of prey sequence output, and thus, has more deviation than the other data sets.

Figure S6. Species accumulation curves for each sampled predator, based on number of samples and detected number of prey species (y-axis). Standard deviation is plotted as boxplots on top of the curve. The lowest row is based on a data from a pilot study, with moderate sampling effort and low number of prey sequence output, and thus, has more deviation than the other data sets.

## Supplemental references

1. Vesterinen, E. J. *et al.* What you need is what you eat? Prey selection by the bat *Myotis daubentonii*. *Molecular Ecology* **25**, 1581–1594 (2016).

2. Zeale, M. R. K., Butlin, R. K., Barker, G. L. A., Lees, D. C. & Jones, G. Taxon-specific PCR for DNA barcoding arthropod prey in bat faeces. *Molecular Ecology Resources* **11**, 236–244 (2011).

3. Clarke, L. J., Czechowski, P., Soubrier, J., Stevens, M. I. & Cooper, A. Modular tagging of amplicons using a single PCR for high-throughput sequencing. *Molecular Ecology Resources* **14**, 117–121 (2014).

4. Clarke, L. J., Czechowski, P., Soubrier, J., Stevens, M. I. & Cooper, A. Modular tagging of amplicons using a single PCR for high-throughput sequencing. *Molecular Ecology Resources* **14**, 117–121 (2014).

5. Vesterinen, E. J., Puisto, A. I. E., Blomberg, A. & Lilley, T. M. Table for five, please: dietary partitioning in boreal bats. *Ecology and Evolution* **8**, 10914–10937 (2018).

6. Vesterinen, E. J., Puisto, A. I. E., Blomberg, A. S. & Lilley, T. M. Data from: Table for five, please: dietary partitioning in boreal bats. *Dryad Dataset* (2019) doi:10.5061/dryad.6880rf1.

7. Kaunisto, K. M., Roslin, T., Sääksjärvi, I. E. & Vesterinen, E. J. Pellets of proof: First glimpse of the dietary composition of adult odonates as revealed by metabarcoding of feces. *Ecology and Evolution* **7**, 8588–8598 (2017).

8. Kaunisto, K. M., Roslin, T. L., Sääksjärvi, I. E. & Vesterinen, E. J. Data from: Pellets of proof: first glimpse of the dietary composition of adult odonates as revealed by metabarcoding of feces. *Dryad Dataset* (2018) doi:10.5061/dryad.5n92p.

9. Kaunisto, K. M. *et al.* Threats from the air: Damselfly predation on diverse prey taxa. *J Anim Ecol* 1365-2656.13184 (2020) doi:10.1111/1365-2656.13184.

10. Vesterinen, E. J. *et al.* Threats from the air: damselfly predation on diverse prey taxa. 1438406240 bytes (2019) doi:10.5061/DRYAD.ZS7H44J4Z.

11. *FASTX Toolkit http://hannonlab.cshl.edu/fastx_toolkit/index.html by Hannon Lab*.

12. Martin, M. Cutadapt removes adapter sequences from high-throughput sequencing reads. *EMBnet.journal* **17**, 10 (2011).

13. Edgar, R. C. Search and clustering orders of magnitude faster than BLAST. *Bioinformatics* **26**, 2460–2461 (2010).

14. Deagle, B. E. *et al.* Counting with DNA in metabarcoding studies: How should we convert sequence reads to dietary data? *Molecular Ecology* **28**, 391–406 (2019).
